# Single‐Cell Hyperthermia: Diamond Quantum Thermometry Reveals Thermal Control of Macrophage Polarization

**DOI:** 10.1002/adma.202517076

**Published:** 2025-12-07

**Authors:** Kaiqi Wu, Qi Lu, Yong Ren, Priyadharshini Balasubramanian, Kazem Ebadi Jalal, Hannah Klug, Matthias Klein, Toszka Bohn, Tobias Bopp, Fedor Jelezko, Yingke Wu, Tanja Weil

**Affiliations:** ^1^ Max Planck Institute for Polymer Research Ackermannweg 10 55128 Mainz Germany; ^2^ Institute for Quantum Optics Ulm University Albert‐Einstein‐Allee 11 89081 Ulm Germany; ^3^ Institute of Immunology University Medical Center of the Johannes Gutenberg University Mainz Langenbeckstraße 1 55131 Mainz Germany; ^4^ Research Center for Immunotherapy (FZI) University Medical Center Mainz 55131 Mainz Germany; ^5^ German Cancer Consortium (DKTK) 69120 Heidelberg Germany; ^6^ University Cancer Center (UCT) Mainz University Medical Center Mainz 55131 Mainz Germany; ^7^ Institute for Quantitative and Computational Biosciences (IQCB) 55128 Mainz Germany; ^8^ Centre for Healthy Ageing Johannes Gutenberg‐University Mainz 55128 Mainz Germany

**Keywords:** fluorescent nanodiamond, immune response modulation, intracellular sensing, lysosomal temperature regulation

## Abstract

Fever elevates body temperature to enhance immune response; however, intracellular temperature can fluctuate by up to 15 °C, suggesting a previously unrecognized layer of thermal regulation. While hyperthermia has long been exploited in medicine, how localized temperature gradients influence cellular fate remains poorly understood. Here, a dual‐function nanodiamond platform is introduced that integrates optically detected magnetic resonance (ODMR) thermometry with croconium‐dye‐based photothermal heating to precisely modulate temperature within endo‐lysosomal compartments of macrophages. Controlled intracellular hyperthermia triggers oxidative stress, transcriptional reprogramming, and polarization toward a pro‐inflammatory phenotype, as confirmed by immunofluorescence, flow cytometry, and transcriptomics. These findings reveal intracellular thermal gradients as active regulators of immune signaling and gene expression. By establishing a direct subcellular thermal trigger for immune activation, independent of the canonical heat‐shock pathway. This work introduces a quantum‐enabled strategy for probing and programming cellular thermodynamics at the nanoscale.

## Graphical abstract

Photothermal nanodiamonds with quantum sensing capabilities generate lysosome‐confined hyperthermia, enabling simultaneous temperature and radical detection via diamond sensing. This localized thermal and oxidative stress drives pro‐inflammatory (M1) macrophage polarization, revealing a new strategy for precision immunomodulation through subcellular control of heat and redox signaling.

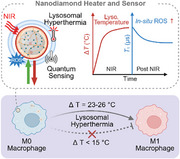

## 1 Introduction

Temperature is a fundamental regulator of cellular function. Even modest changes in core body temperature, such as those occurring during fever, can profoundly affect immune surveillance and pathogen clearance.^[^ ^1^, ^2^ ^]^ While systemic hyperthermia is well established in host defense and clinical therapy, emerging evidence reveals that intracellular temperature can vary far more dramatically. Notably, mitochondria have been shown to reach temperatures up to 15 °C above the surrounding cytoplasm,^[^ ^3^, ^4^ ^]^ suggesting that localized thermal gradients could represent an unrecognized layer of cellular regulation.^[^ ^5^, ^6^ ^]^

Thermal modulation has long been exploited in medicine, from clinical hyperthermia (40–43 °C) that sensitizes tumors to radio‐ and chemotherapy^[^ ^7^ ^]^ to thermal ablation (> 50 °C) that induces rapid coagulative necrosis.^[^ ^8^ ^]^ Beyond tissue‐level interventions, localized photothermal heating at the cellular level or even the organelle level^[^ ^9^, ^10^ ^]^ has been reported, underscoring the potential for spatially confined thermal modulation in living systems. Organelle‐specific perturbations, such as lysosomal^[^ ^11^ ^]^ or mitochondrial^[^ ^12^ ^]^ stress, are now recognized as key regulators of immune and metabolic signaling pathways. However, the magnitude and spatial distribution of heat generated within cells or organelles and how such compartmentalized temperature elevations influence signaling remain poorly understood. Addressing these questions requires tools that can both measure and manipulate thermal gradients within defined subcellular compartments, capabilities that remain beyond conventional fluorescent thermosensors.

To contextualize this challenge, we benchmarked representative biomedical thermometry techniques (Table S1, Supporting Information), comparing their spatial resolution, temperature sensitivity, and photostability. Fluorescence‐based probes such as rhodamine B, europium complexes, green fluorescent proteins, and quantum dots suffer from photobleaching and environmental cross‐sensitivity.^[^ ^13^, ^14^ ^]^ Moreover, their spectral overlap with photothermal actuators complicates simultaneous heating and sensing.

Fluorescent nanodiamonds (FNDs) containing nitrogen‐vacancy (NV^−^) centers^[^ ^15^, ^16^, ^17^ ^]^ offer a fundamentally different approach. Their optically detected magnetic resonance (ODMR) enables photostable, quantitative temperature readouts with sub‐degree precision and nanometric resolution, while NV‐based *T*_1_ relaxometry detects local paramagnetic radicals in real time, have emerged as powerful and robust quantum sensors capable of probing physical and chemical parameters, including temperature variations,^[^ ^18^, ^19^, ^20^, ^21^ ^]^ magnetic field,^[^ ^22^, ^23^, ^24^ ^]^ pH shifts,^[^ ^25^, ^26^ ^]^ paramagnetic species^[^ ^27^, ^28^, ^29^ ^]^ at the nanoscale with exceptional photostability and biocompatibility. ODMR thermometry, first demonstrated in living cells,^[^ ^19^ ^]^ has since been extended to multicellular organisms such as *C. elegans* ^[^ ^20^, ^30^ ^]^ and neurons.^[^ ^31^ ^]^ Recent efforts have improved sensitivity,^[^ ^32^ ^]^ and enabled intracellular temperature gradient sensing.^[^ ^4^, ^33^ ^]^ When integrated with photothermal agents, this platform allows simultaneous quantum‐based temperature sensing and localized heating.

**Figure 1:**
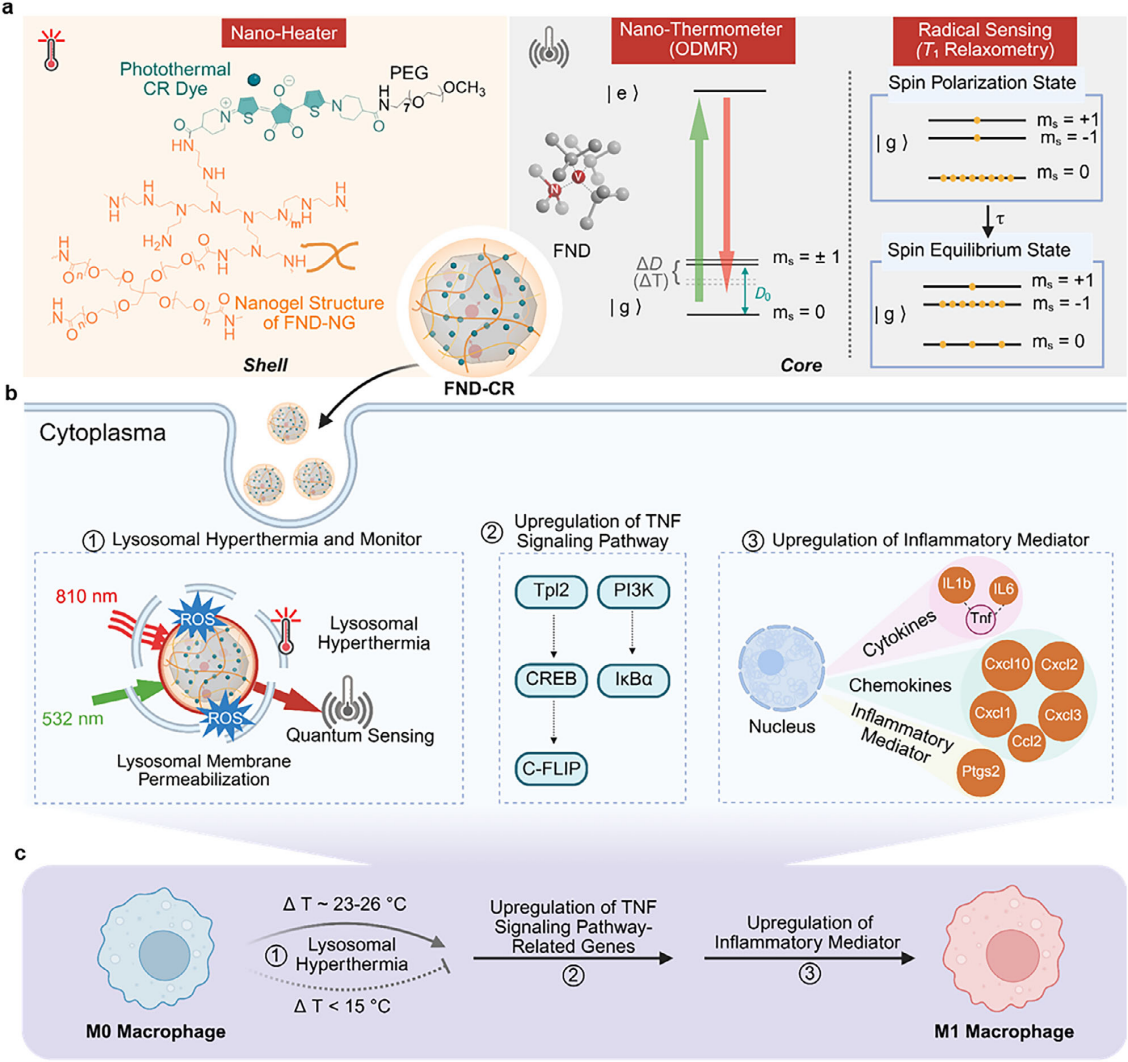
Schematic of Multifunctional fluorescent nanodiamonds for intracellular thermometer and heating drives pro‐inflammatory polarization of macrophages. a) Fluorescent nanodiamonds (FND) decorated with croconium (CR) dyes (FND‐CR) serve as both intracellular nano‐heaters and quantum sensors. Photothermal CR dyes embedded in the nanogel layer generate heat upon near‐infrared (NIR, 810 nm) irradiation. The FND core contains nitrogen‐vacancy (NV^−^) centers that enable nanoscale temperature sensing via optically detected magnetic resonance (ODMR), where temperature‐dependent zero‐field splitting (Δ*D*) between spin sublevels |*m_s_* = 0⟩ and |*m_s_* = ±1⟩ is used for thermal readout. Radical sensing is performed via *T*_1_ relaxometry, which detects local paramagnetic species based on spin relaxation dynamics between spin sublevels |*m_s_* = 0⟩ and |*m_s_* = ±1⟩. b) NIR‐induced lysosomal heating triggers oxidative stress and lysosomal membrane permeabilization (LMP) (①), leading to the upregulation of tumor necrosis factor (TNF) signaling pathway‐related genes, including Tpl2 and PI3K (②). This, in turn, drives the transcriptional upregulation of pro‐inflammatory (M1) mediators such as *Ptgs2, Il1b, Il6, and Cxcl10* (③). c) Localized lysosomal hyperthermia ultimately drives macrophage polarization from the M0 toward the pro‐inflammatory M1 state. Figure 1 created with BioRender.com.

Here, we introduce croconium‐decorated fluorescent nanodiamonds (FND‐CR) as multi‐functional quantum sensors that unite local photothermal actuation, quantum thermometry, and radical detection in a single platform. Croconium dyes provide strong near‐infrared absorption, high photothermal conversion efficiency, and excellent photostability,^[^ ^34^, ^35^, ^36^ ^]^ outperforming conventional agents such as indocyanine green. Targeted to lysosomes in macrophages, FND‐CR enables organelle‐specific heat generation and real‐time temperature monitoring via ODMR, and in situ detection of radicals via *T*_1_ relaxometry (**Figure** **1**). We reveal that localized lysosomal hyperthermia induces oxidative stress, transcriptional reprogramming, and macrophage polarization toward a pro‐inflammatory (M1) phenotype. These findings establish subcellular temperature as a programmable biophysical cue and demonstrate a quantum‐enabled strategy for dissecting the thermal control of immune function.

## 2 Results and Discussion

### 2.1 FND‐CR Generate Heat and Serve as Quantum Nanothermometer

Water‐soluble, photostable CR dyes were synthesized as described previously^[^ ^34^ ^]^ and fully characterized (Figures S1 and S3–S6, Supporting Information). The dyes were covalently conjugated to nanogel‐coated fluorescent nanodiamonds (FND‐NG)^[^ ^37^ ^]^ via amide coupling (Figures S2,S7 and S8 and Table S2, Supporting Information). The resulting nanoparticle conjugate, FND‐CR, integrates near‐infrared photothermal localized heating with temperature sensing through NV^−^ centers of FND in living cellular environments (Figure 1).

**Figure 2:**
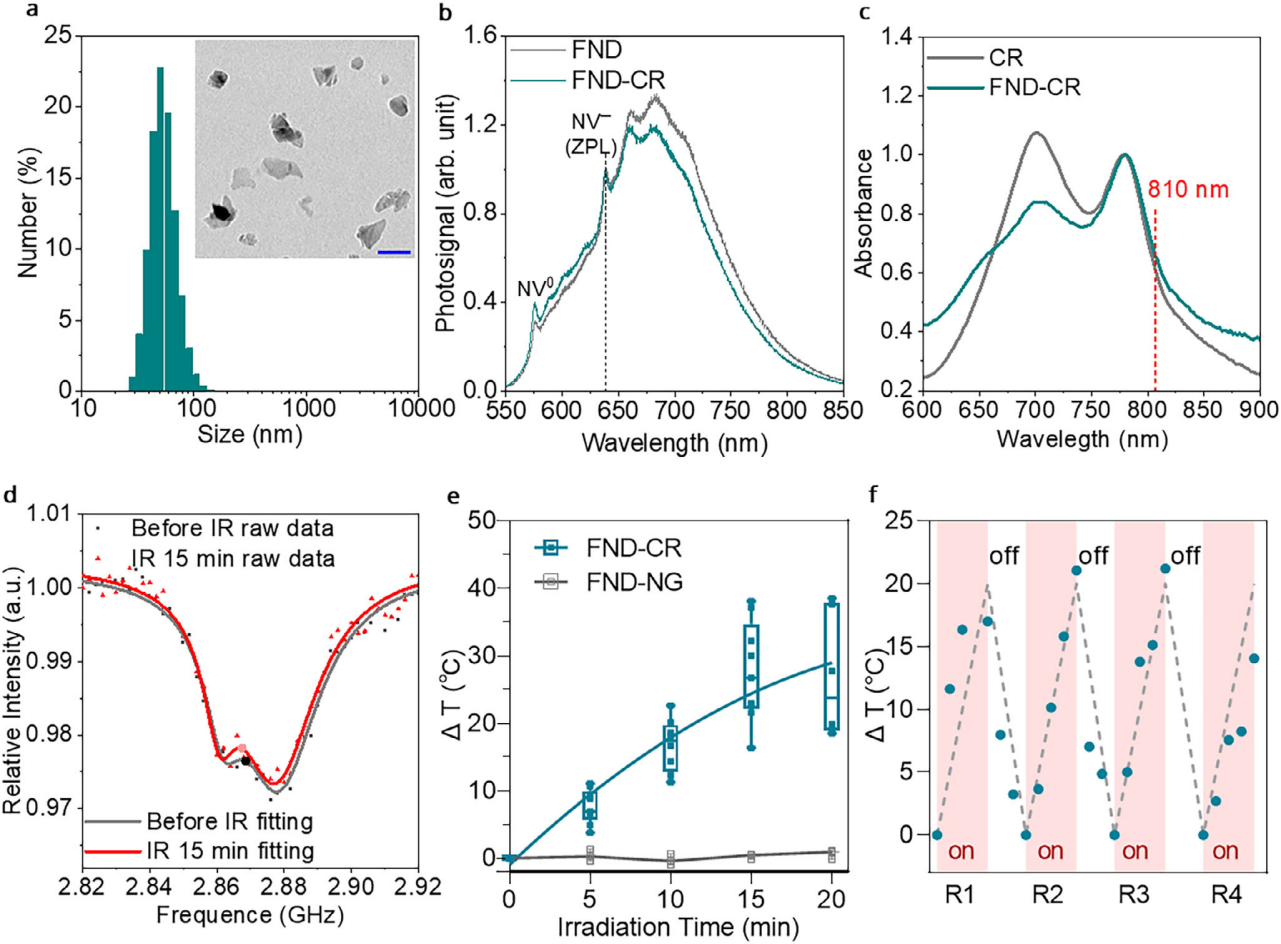
Characterization of the FND‐CR nano‐heaters and quantum thermometers. a) Size distribution profile of FND‐CR, as measured by dynamic light scattering (DLS). Inset: Transmission electron microscopy (TEM) image of FND‐CR, scale bar: 50 nm. b) Fluorescence spectra of FND and FND‐CR. The zero‐phonon line at 637 nm remained intact after CR‐PEG conjugation. c) Visible‐near infrared (Vis‐NIR) spectra of CR and FND‐CR, normalized to absorbance at 782 nm. d) Representative ODMR spectra of FND‐CR before and after NIR irradiation (810 nm, 0.6 W cm^−^^2^). e) Temperature change measured by ODMR on single FND‐CR particles under varying durations of NIR irradiation (810 nm, 0.6 W cm^−^^2^) (n = 7). FND‐NG particles without CR were used as a negative control (n = 3). The temperature baseline was assumed as room temperature (22 °C). f) Temperature change measured by ODMR in a single FND‐CR particle across 4 rounds of NIR irradiation (810 nm, 0.6 W cm^−^^2^). Each cycle consisted of 15 min of heating followed by 15 min of cooling, with temperature recorded every 5 min during the process.

Dynamic light scattering (DLS) analysis indicated a hydrodynamic diameter of 55 ± 4 nm for FND‐CR. No aggregation was observed in solution, and transmission electron microscopy (TEM) confirmed the presence of well‐dispersed individual nanoparticles without detectable aggregation (**Figure** **2**a). Because the charge state and optical properties of NV^−^ centers in FNDs are sensitive to surface modifications, we evaluated whether CR conjugation affects their quantum sensing functionality. Emission spectra of FND‐CR retained a distinct zero‐phonon line (ZPL) at 637 nm (Figure 2b), indicating that the NV^−^ centers remain optically active and compatible with optically detected magnetic resonance (ODMR)–based thermometry. FND‐CR also exhibited a broad near‐infrared (NIR) absorption spectrum with characteristic peaks at 700 and 782 nm, consistent with the spectral signature of CR dyes and confirming successful conjugation (Figure 2c). Visible‐ near infrared (Vis‐NIR) spectroscopy quantified the dye loading at 8.6 µg of CR per 100 µg of FND‐NG, corresponding to ≈1000 CR molecules per FND‐CR particle (Figure S9, Supporting Information).

To evaluate the photothermal response of individual FND‐CR, we performed ODMR spectroscopy at the single‐nanoparticle level. A linear gold microwave antenna was lithographically patterned onto glass coverslips for uniform microwave delivery, following the protocol established by Oshimi et al.,^[^ ^30^ ^]^ FND‐CR were immobilized on the substrate, and ODMR spectra were acquired under continuous green laser excitation (532 nm, 20 µW) while sweeping the microwave frequency from 2.82 to 2.92 GHz (−25 dBm, decibels relative to one milliwatt). Upon NIR irradiation at 810 nm (0.6 W cm^−^^2^) for 15 min, a downshift in the zero‐field splitting parameter (*D*_0_) was observed (Figure 2d), indicating an increase in the local temperature within the diamond lattice. The corresponding temperature change (Δ*T*) was calculated from the shift of the zero‐field splitting using the following equation:

(1) $$\Delta T=\frac{\Delta D}{\alpha }$$

where Δ*D* represents the shift in the transition frequency, and α = −74 kHz/K is the temperature sensitivity coefficient.^[^ ^38^ ^]^

To probe the nanoscale photothermal dynamics of FND‐CR, ODMR spectra were recorded at 5‐min intervals over 20 min of continuous NIR irradiation (810 nm) at 0.6 W cm^−^^2^. A steady increase in local temperature was observed during the first 15 min, reaching a peak increase Δ*T* of 27.5 ± 6.9 °C starting from the room temperature (22 °C as the baseline), followed by a plateau, indicative of thermal equilibrium between heat generation and dissipation. In contrast, FND‐NG lacking CR conjugation exhibited no significant temperature changes under irradiation (Figure 2e), confirming that CR irradiation induced a photothermal response. The photothermal behavior of FND‐CR was further evaluated in aqueous dispersion (0.1 mm CR equivalent) using conventional thermal imaging. Upon NIR irradiation, the solution temperature increased by ≈17 °C above baseline within 8 min and then plateaued (Figure S10a, Supporting Information). Notably, FND‐CR exhibited consistent photothermal behavior across four consecutive irradiation cycles (810 nm, 0.6 W cm^−^^2^), both measurements performed at the single‐particle level in the dry state (Figure 2f) and in aqueous solution (Figure S10b, Supporting Information), highlighting its high photostability and reusability.

### 2.2 FND‐CR Generate Localized Lysosomal Hyperthermia, Monitor Temperature, and Sense Oxidative Stress In Situ in J774A.1

**Figure 3:**
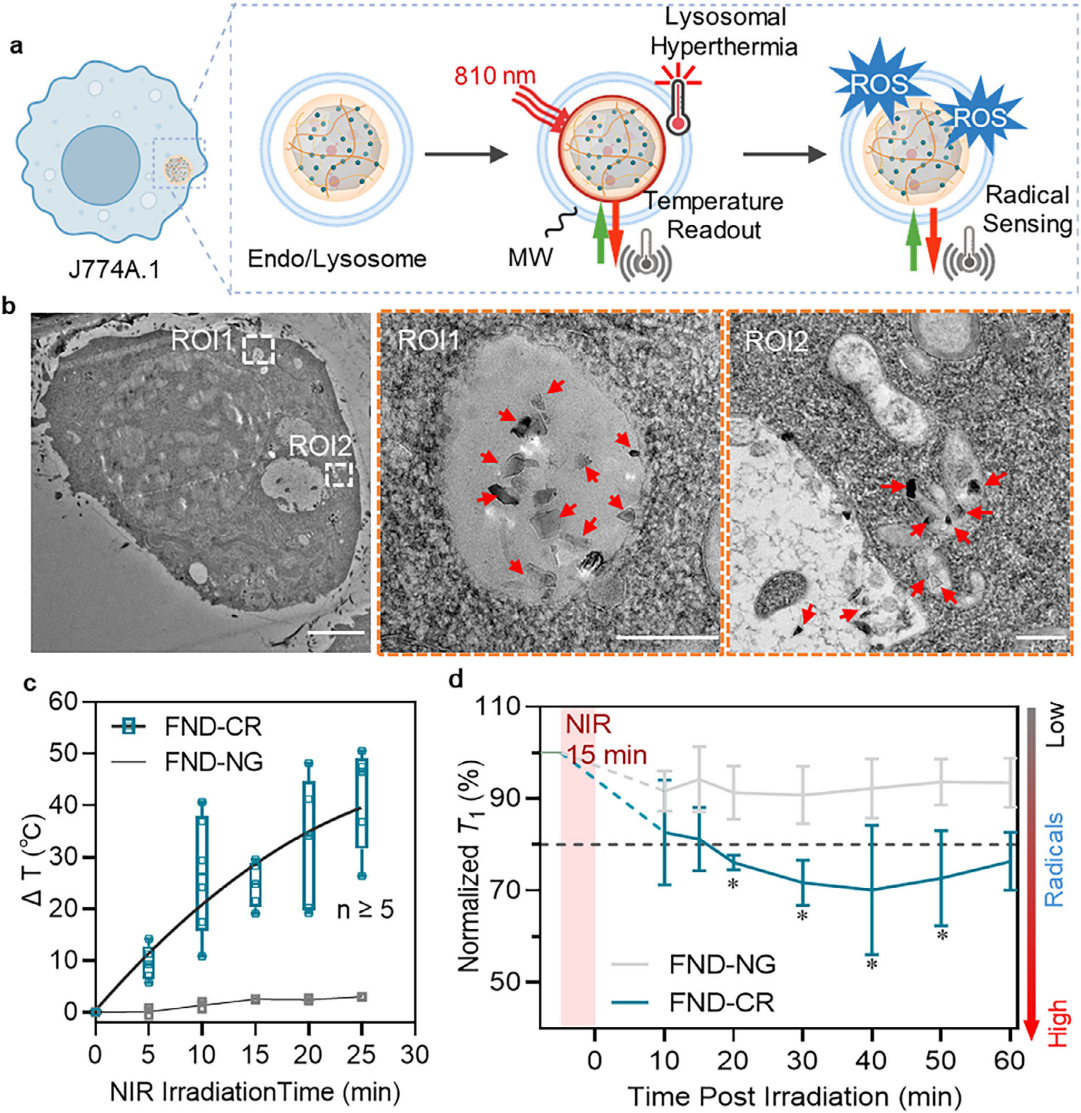
FND‐CR accumulates in lysosomal vesicles, enabling localized heating, quantum temperature sensing, and in situ radical detection. a) Schematic illustration of the intracellular localization of FND‐CR nanoparticles in J774A.1 macrophage. Upon NIR irradiation, the photothermal CR dyes generate localized lysosomal heat, which is monitored via quantum sensing (via ODMR) by NV^−^ centers and further induces localized oxidative stress detected by *T*_1_ relaxometry‐based radical sensing. b) Transmission electron microscopy of J774A.1 cell shows internalized FND‐CR particles after 12 h of incubation at 37 °C. Images with orange dashed borders represent the zoomed‐in areas. Dashed boxes indicate magnified regions; red arrows highlight FND‐CR particles. Scale bars: full image, 2 µm; zoom‐in, 200 nm. c) Intracellular temperature change readout from ODMR measurements on individual FND‐CR particles under NIR irradiation (810 nm, 0.6 W cm^−^^2^) for 25 min (n ≥ 5). FND‐NG particles without CR dyes served as a negative control (n = 3). The temperature baseline was assumed as room temperature (22 °C). d) In situ radical sensing via time‐resolved *T*_1_ relaxometry on individual FND‐CR particles. *T*_1_ relaxation time was measured continuously for 10 min before irradiation, paused during the 15‐min irradiation, and resumed afterward to monitor intracellular radical dynamics. FND‐NG (without CR) served as a control. A decrease in *T*_1_ time indicates an elevated intracellular radical load. Data are represented as the mean ± SD (n = 4); ^*^ *p* < 0.05, one‐way ANOVA.

Murine macrophage‐like J774A.1 cells, originally derived from a mouse reticulum cell sarcoma^[^ ^39^ ^]^ were selected for our study. Their unstimulated, basal state is generally considered to be in an unpolarized M0‐like state that can be polarized experimentally into the pro‐inflammatory M1 or the anti‐inflammatory, tumor‐promoting M2 phenotype.^[^ ^40^ ^]^ FND‐CR particles were internalized and localized within vesicles of J774A.1 macrophages after 12 h of incubation, demonstrated by confocal microscopy and TEM (**Figure** **3**a,b; Figure S11, Supporting Information). For precise intracellular temperature measurements via ODMR, macrophages were cultured on coverslips embedded with gold antennas for microwave loading (Figure S12a,b, Supporting Information). Upon NIR (810 nm) irradiation at 0.6 W cm^−^^2^, individual FND‐CR particles exhibited a rapid temperature increase of 24.6 ± 3.9 °C within 15 min (Figure 3c). Prolonged irradiation (another 10 min) led to a further ∼10 °C rise, albeit with increased heterogeneity in particle temperature measurement due to enhanced intracellular mobility and defocusing effects. The observed thermal kinetics followed an exponential trend (R^2^ = 0.92), described by the equation

(2) $$\Delta T=a(1-e^{-bt})$$

where *a* represents the maximum temperature change, *b* is the heating rate constant, and *t* is the irradiation time.^[^ ^41^ ^]^

Furthermore, when the irradiation power was reduced by half (0.3 W cm^−^^2^), the observed temperature increase was only 11.4  ±  2.7 °C within 15 min and therefore significantly lower (Figure S12c, Supporting Information). These results demonstrate that photothermal‐induced temperature changes within vesicles of living cells scale with laser power, confirming the capability of FND‐CR to precisely induce localized lysosomal hyperthermia.

In addition to the thermal readout, NV^−^ centers allow monitoring of the intracellular radical response upon localized lysosomal heating based on *T*_1_ relaxometry.^[^ ^27^, ^42^ ^]^ The principle of *T*_1_ relaxometry and the corresponding laser pulse sequences used in this study are illustrated in Figure S13a,b (Supporting Information). The presence of paramagnetic species, such as superoxide, nitric oxide, and hydroxyl radicals, accelerates NV^−^ spin relaxation, leading to shortened *T*_1_ times. The sensitivity of FND‐CR toward local radicals was first probed and validated by titration with paramagnetic gadolinium ions (Gd^3+^) outside cells, confirming its responsiveness of FND‐CR to radical‐induced spin noise (Figure S13c, Supporting Information). Following NIR irradiation, *T*_1_ relaxation times of FND‐CR decreased significantly, which indicates the radical generation from 20 to 50 min post‐irradiation (Figure 3d). In contrast, FND‐NG, lacking CR conjugation, exhibited no significant changes in *T*_1_ (Figure 3d).

To validate the quantum‐sensing results using a conventional assay, intracellular reactive oxygen species (ROS) were quantified with 2′,7′‐dichlorodihydrofluorescein diacetate (DCFH‐DA) staining. Consistent with the *T*_1_ relaxometry data, cells treated with FND‐CR and subjected to NIR irradiation (810 nm, 0.6 W cm^−^^2^) exhibited a marked increase in fluorescence intensity (mean value rising from 5.86 ± 1.34 to 9.50 ± 4.00 a.u.), confirming elevated oxidative stress (Figure S14, Supporting information). In contrast to the dynamic, organelle‐resolved readout provided by quantum relaxometry, the DCFH‐DA assay offers only a static, irreversible snapshot of total ROS and lacks temporal or subcellular specificity.^[^ ^43^ ^]^

Collectively, these complementary results demonstrate that lysosomal heating induces both localized and mild global oxidative stress. The time‐resolved radical dynamics of radical formation provide direct mechanistic evidence that localized lysosomal hyperthermia not only elevates vesicular temperature but also generates in situ intracellular oxidative stress, potentially contributing to downstream immune activation.^[^ ^44^, ^45^ ^]^

### 2.3 Localized Lysosomal Hyperthermia Drives Vesicles‐Related Transcriptional Reprogramming of Macrophages

Macrophages display remarkable functional plasticity, sensing extra‐and intracellular changes and balancing pro‐inflammatory responses with tissue repair properties through diverse phenotypes.^[^ ^46^, ^47^ ^]^ They can differentiate into specialized functional phenotypes, adopting characteristic immune functions in response to microenvironmental cues.^[^ ^48^ ^]^ While extracellular factors influencing macrophage polarization have been extensively characterized,^[^ ^49^ ^]^ the contribution of intracellular temperature remains largely unexplored. Notably, cells typically undergo thermal damage near 45 °C, a threshold often exploited in photothermal cancer therapy.^[^ ^50^ ^]^ In this study, J774A.1 macrophages in the resting, unpolarized, and undifferentiated M0 state tolerated localized endo‐lysosomal heating up to 46.6  ±  3.9 °C (localized lysosomal hyperthermia) following 15 min of NIR irradiation without significant cytotoxicity, as confirmed by apoptosis assays (Figures S15 and S16, Supporting Information). It should be noted that this intracellular temperature gradient represents a localized, photothermally induced hyperthermia generated by FND‐CR nanoparticles, rather than a naturally occurring physiological temperature rise. To investigate the transcriptional responses to lysosomal hyperthermia, the total RNA was extracted from treated J774A.1 cells and subjected to whole‐transcriptome analysis. Interestingly, transcriptome analysis revealed that classical heat shock proteins were not upregulated following lysosomal heating (Figure S17a,b, Supporting Information). This absence of canonical heat shock activation supports that temperature elevation was spatially confined to endo‐lysosomal compartments and insufficient to trigger global cytosolic heat stress responses.

**Figure 4:**
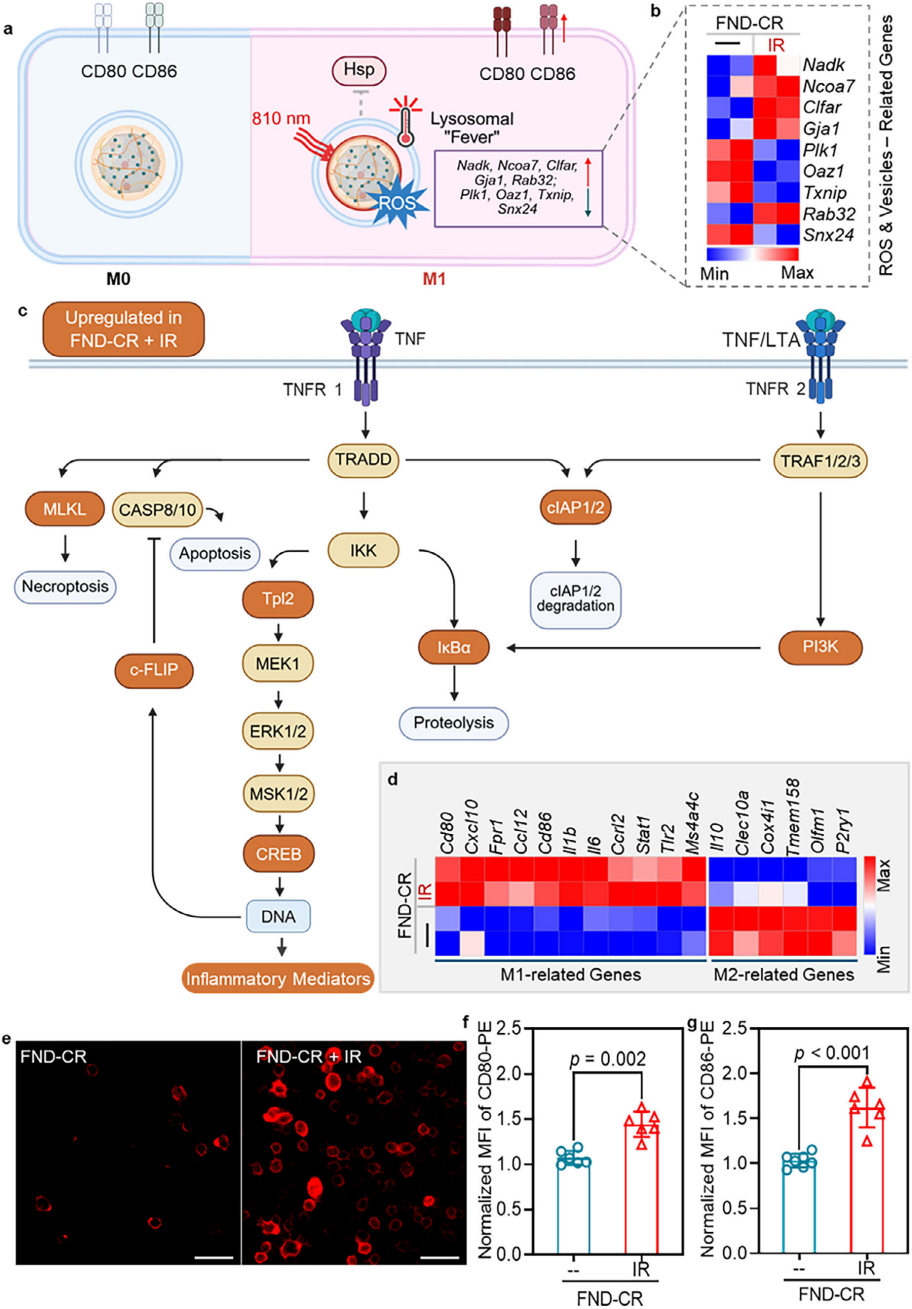
FND‐CR‐induced lysosomal hyperthermia drives pro‐inflammatory (M1) polarization and transcriptional reprogramming in J774A.1 macrophage. a) Schematic illustration of endo‐lysosomal hyperthermia generated by FND‐CR under NIR irradiation (810 nm, 0.6 W cm^−^^2^), leading to localized oxidative stress, which drives macrophages toward the pro‐inflammatory M1 phenotype. b) Transcriptome heatmap showing expression (Fold Change > 2, p‐value < 0.05) genes associated with cellular oxidative stress, lysosomal integrity, and vesicle trafficking following FND‐CR‐mediated heating. c) Comparative enrichment analysis of the TNF‐α pathway of FND‐CR under NIR irradiation (0.6 W cm^−2^) versus FND‐CR alone. d) Heatmap from transcriptome profiling highlighting the expression of genes associated with M1 and M2 macrophage polarization (Fold Change > 2, p‐value < 0.05). e) Representative fluorescence images of J774A.1 macrophages co‐stained with anti‐CD80‐PE and anti‐CD86‐PE antibodies 20 h after NIR irradiation (810 nm, 0.6 W cm^−^^2^) following FND‐CR uptake. Scale bar, 50 µm. f,g) Quantification of mean fluorescence intensity (MFI) of anti‐CD80‐PE (f) and anti‐CD86‐PE (g) stained macrophages, obtained from flow cytometry analysis (n = 6). MFI values were normalized to the values of the untreated control group. An unpaired *t‐test* was used to calculate statistical significance, with *p*‐values indicated.

Instead, localized lysosomal hyperthermia triggered distinct vesicle‐associated stress responses. Transcriptomic profiling revealed significant upregulation of genes associated with vesicular repair (*Ncoa7*), membrane trafficking (*Rab32*), redox buffering (*Nadk*), and oxidative stress responses (*Gja1*) (**Figure** **4**a,b; Table S3, Supporting Information). These changes in expression are consistent with an adaptive response to localized ROS accumulation, also detected by radical sensing via *T*_1_ relaxometry (Figure 3d). Consistent with transcriptomic profiling, transmission electron microscopy revealed partial loss or discontinuity of vesicular membranes following irradiation, indicative of localized lysosomal membrane rupture in a subset of vesicles (Figure S18, Supporting Information). These structural changes further support our hypothesis that localized lysosomal heating can compromise vesicle integrity and contribute to downstream inflammatory signaling.^[^ ^11^, ^51^ ^]^

### 2.4 Localized Lysosomal Hyperthermia Regulates Transcriptional Changes in Macrophage Polarization

Macrophage polarization into M1 (pro‐inflammatory) or M2 (anti‐inflammatory) states requires extensive transcriptional reprogramming, driven by master regulators. For example, M1 polarization is fostered by activation of transcription factors such as NF‐κB, STAT1, and IRF5, which upregulate pro‐inflammatory genes (e.g., TNF‐α and iNOS) and catabolic pathways.^[^ ^52^ ^]^ In response to localized lysosomal hyperthermia, pathway enrichment analysis revealed significant activation of tumor necrosis factor‐alpha (TNF‐α) signaling modules (Figure 4c; Figure S19a, Supporting Information). Upregulated components, including *Map3k8 (Tpl2)*, *Cflar (c‐FLIP)*, *Birc2/3*, and *Creb1* (see detailed functions of these genes in Figure S19, Supporting Information), are consistent with activation of pro‐inflammatory and non‐canonical NF‐κB signaling and survival pathway engagement.^[^ ^53^, ^54^, ^55^, ^56^ ^]^ These transcriptional changes suggest that localized lysosomal hyperthermia integrates oxidative stress and vesicular stress with cytokine receptor signaling to shift macrophages toward a pro‐inflammatory M1 fate (Figure S19b, Supporting Information).

Consistent with the engagement of TNF‐α signaling pathways, transcriptome profiling further confirmed M1 reprogramming, with robust upregulation of canonical pro‐inflammatory genes including *Cd80*, *Cd86*, *Il6*, *Il1b*, and *Cxcl10*, accompanied by downregulation of M2 genes such as *Il10, Clec10a, Cox4i1, Olfm1*, and *P2ry1* (Figure 4d). By producing these pro‐inflammatory cytokines, presenting antigens, and exerting cytotoxic effects, M1 macrophages play a pivotal role in anti‐tumor immune responses.^[^ ^40^ ^]^ Beyond the transcriptional shifts, this polarization was phenotypically confirmed at the protein level by immunofluorescence staining and flow cytometry, which demonstrated significant upregulation of CD80 and CD86 surface markers following FND‐CR‐mediated lysosomal heating under NIR irradiation (0.6 W cm^−^^2^), whereas FND‐CR alone did not elicit detectable polarization (Figure 4e–g; Figures S20–S22, Supporting Information).

To assess the temperature dependence of this polarization, irradiation power was reduced by half (0.3 W cm^−^^2^), resulting in a vesicular temperature rise of only 11.4  ±  2.7 °C (Figure S12c, Supporting Information). Under these conditions, no significant upregulation of CD80 or CD86 expression was observed (Figures S20–S22, Supporting Information), suggesting that a critical intracellular temperature threshold must be exceeded to drive M1 polarization. Together, these results demonstrate that compartmentalized lysosomal hyperthermia is sufficient to reprogram macrophages into a pro‐inflammatory phenotype through temperature‐sensitive transcriptional and signaling pathways.

To further verify the role of oxidative stress in lysosomal‐heat–induced M1 macrophage polarization, radicals generated during lysosomal hyperthermia were scavenged using 5 mm N‐acetyl‐L‐cysteine (NAC). NAC treatment markedly reduced both global and lysosome‐specific oxidative stress following FND‐CR‐mediated heating (Figure S23a–c, Supporting Information), confirming its efficacy in mitigating ROS accumulation. Correspondingly, NAC treatment significantly attenuated the upregulation of CD80 and CD86 expression (Figure S24, Supporting Information), demonstrating that oxidative stress contributes critically to the activation of the pro‐inflammatory phenotype. However, ROS scavenging did not fully reverse macrophage polarization, as CD86 expression remained modestly elevated compared with the non‐irradiated FND‐CR group (Figure S24, Supporting Information). This suggests that, while oxidative stress is a key mediator of lysosomal heat‐induced immune activation, additional temperature‐sensitive signaling mechanisms may also be involved. As a reference, NAC has previously been reported to transiently suppress LPS‐induced macrophage activation within 6 h,^[^ ^57^, ^58^ ^]^ but not after prolonged stimulation (20 h, Figure S25, Supporting Information).

### 2.5 Perspectives on Primary Cells

While the J774A.1 cell line provides a robust and reproducible model for dissecting thermal control of polarization, primary bone marrow–derived macrophages (BMDMs) offer a more physiologically relevant system for assessing translational potential. To examine whether lysosomal hyperthermia elicits similar immune responses in primary macrophages, BMDMs were subjected to identical FND‐CR treatment and NIR irradiation conditions. A similar trend toward M1‐like activation was detected, with increased CD80/CD38 expression in selected biological replicates (Figure S26a, Supporting Information). However, the overall response was attenuated and more variable, with pronounced polarization observed in only two out of five independent experiments (Figure S26b, Supporting Information). ODMR‐based thermometry revealed that the maximal lysosomal temperature rise in BMDMs was ≈8–12 °C lower than in J774A.1 cells under identical irradiation durations (15–20 min, Figure S27, Supporting Information), indicating that the threshold temperature required for robust polarization was not consistently reached. This reduced heating efficiency likely reflects differences in metabolic heat dissipation, vesicular composition, and stress tolerance between primary and immortalized macrophages. They also emphasize that macrophage activation cannot be fully captured by a limited M1/M2 marker panel, given the broad diversity of macrophage phenotypes.

Together, these findings validate the underlying concept of lysosomal hyperthermia as a thermal cue for immune modulation, while emphasizing the need for optimized photothermal coupling and irradiation parameters in primary and in vivo models. The results thus establish a proof‐of‐concept framework for controlled subcellular thermomodulation and open pathways toward exploring thermal signaling in physiologically relevant immune contexts.

## 3 Conclusion

By engineering a multifunctional nanodiamond platform that unites precise thermal control with quantum sensing, we uncover that intracellular thermal gradients, manifested as *localized lysosomal hyperthermia*, can actively dictate immune cell fate. Controlled lysosomal heating not only elevates vesicular temperature but simultaneously generates oxidative stress, detected in situ through quantum relaxometry. These compartmentalized stress responses surpass the canonical heat‐shock paradigm, establishing a direct thermal threshold for macrophage polarization toward a pro‐inflammatory phenotype. This study introduces a quantum‐enabled approach to manipulate and decode subcellular thermodynamics, positioning artificially induced hyperthermia as a programmable biological signal rather than a passive consequence of metabolism. The ability to correlate temperature, radical flux, and immune activation within a single nanoplatform defines a new strategy for probing the causal links between heat and cell function. Beyond its immediate biological insight, this work provides a foundational framework for precision thermobiology and immune engineering, where temperature can be harnessed as a controllable input to regulate signaling pathways and cell behavior.

We envision that integrating additional NV‐based modalities, such as magnetic resonance–based detection of paramagnetic ions^[^ ^59^, ^60^ ^]^ or pH‐dependent surface charge modulation^[^ ^26^ ^]^ will enable multiplexed mapping of subcellular environments. While current implementations of FND‐based sensing rely on specialized optical instrumentation,^[^ ^61^, ^62^ ^]^ rapid advances in widefield ODMR imaging and portable quantum diamond microscopes (e.g., Qnami, QDTI, anQT Sense) are expanding accessibility and throughput. Combined with localized photothermal actuation, these developments will transform NV‐center nanodiamonds into versatile, next‐generation tools for multimodal biosensing, intracellular diagnostics, and heat‐driven therapeutic modulation.

## 4 Experimental Section

### Preparation of Photothermal Nanodiamond Heater and Quantum Sensor (FND‐CR)

Fluorescent nanodiamond nanogel (FND‐NG) containing primary amino groups was prepared as described in the previous study.^[^ ^37^ ^]^ Briefly, 40 µL of freshly prepared 4‐arm polyethylene glycol‐succinimidyl carbonate (PEG‐SCM, MW: 10 kDa, 2.0 mg) aqueous solution was added to a mixture of 200 µL of 1 mg mL^−1^ FND aqueous solution, 100 µL of 20 mg mL^−1^ polyvinylpyrrolidone (PVP, MW: 10 kDa), 200 µL of 5 mg mL^−1^ polyethyleneimine (PEI, MW: 25 kDa), and 300 µL of PBS. The mixture was placed on a shaker at 800 rpm at room temperature for 6 h. The formed FND‐NG was collected by centrifugation at 14 000 rpm for 30 min and washed twice with Milli‐Q water.

Subsequently, mPEG‐CR was conjugated to FND‐NG via N‐ethyl‐N'‐(3‐dimethylaminopropyl)‐carbodiimide (EDC)/N‐hydroxysuccinimide (NHS) coupling chemistry. To this end, 200 µL of freshly prepared EDC/NHS solution (0.8/0.6 mg, 4.0/5.1 µmol) in cold aqueous solution was added to 0.1 mg of mPEG‐CR dissolved in 1 mL of a DMF/H_2_O mixture (1:4, v/v). The reaction mixture was stirred on ice for 15 min, followed by an additional 15 min at room temperature. Next, 200 µL of 1 mg mL^−1^ FND‐NG aqueous solution was added to the reaction mixture, stirring overnight at room temperature. The final FND‐CR complex was collected by centrifugation at 14 000 rpm for 30 min and washed three times with Milli‐Q water.

### Characterization of FND‐CR

The conjugation efficiency of CR to FND‐NG was determined using the Lambert‐Beer law, based on the UV–vis absorption spectrum of CR. Solutions of CR at varying concentrations (100, 50, 30, 20, 10, 5, and 1 µg mL^−1^) and 20 µL of a 0.5 mg mL^−1^ FND‐CR solution were added to a 384‐well plate. The absorbance between 550 and 900 nm was measured using a plate reader (Tecan Spark 20 m). A calibration curve of absorbance intensity versus CR concentration was generated, which was then used to calculate the CR content in the FND‐CR conjugate. The size of FND‐CR was characterized by both transmission electron microscopy (TEM) and dynamic light scattering (DLS). Specifically, one drop of a 0.1 mg mL^−1^ FND‐CR solution in Milli‐Q water was placed onto an oxygen plasma‐treated copper grid. Bright‐field images were acquired using a JEOL 1400 transmission electron microscope. For DLS measurement, 500 µL of 20 µg mL^−1^ solutions of FND, FND‐NG, or FND‐CR in Milli‐Q water were transferred to a borosilicate glass cuvette and measured at 25 °C with a 90° angle using a particle sizer (Zetasizer Nano Z (Malvern Panalytical)). The hydrodynamic diameter was presented as a number distribution. Zeta potential was measured at 25 °C with a Zetasizer Nano Z (Malvern Panalytical).

### Subcellular Location of FND‐CR in J774A.1

The location of FND‐CR in J774A.1 was characterized by both confocal microscopy (CLSM) and TEM. For the CLSM assay, J774A.1 cells were seeded at a density of 20 000 cells per well in an 18‐well plate (ibidi) one day before treatment. The following day, cells were incubated with either 20 µg mL^−1^ of FND‐CR or 10 µg mL^−1^ of FND‐NG for 14 h at 37 °C in 5% CO_2_. After incubation, cells were washed three times with DPBS, then stained with 50 nM Lysotracker‐Green (ThermoFisher Scientific) for 30 min at 37 °C in 5% CO_2_. Cells were washed again with DPBS three times, re‐supplied with Leibovitz's L‐15 Medium, and imaged directly using a laser confocal microscope (Stellaris 8, Leica) with a 40× magnification objective (NA = 1.25, glycidol immersion). The fluorescence signals from FND‐CR and FND‐NG were detected at excitation/emission wavelengths of 550/700–800 nm, while stained lysosomes were visualized using 504/514–540 nm excitation/emission wavelengths. Additionally, the fluorescence lifetimes of FND‐CR and FND‐NG inside and outside cells were measured using fluorescence lifetime imaging microscopy (FLIM) on a Stellaris 8 system (Leica) to confirm the presence of diamond nanoparticles.

For the TEM assay, J774A.1 cells were cultured in a 24‐well plate, with carbon‐coated sapphire discs (3 mm diameter) pre‐placed at a cell density of 50 000 cells mL^−1^. After co‐incubation with FND‐CR and with NIR irradiation (0.6 W cm^−2^) for 15 min, the sapphire discs were sandwiched between two aluminum plates and mounted into a holder (Engineering Office, M. Wohlwend). The samples were immediately subjected to high‐pressure freezing using a Wohlwend HPF Compact 01 freezer (Engineering Office, M. Wohlwend) at 2100 bar. The frozen samples were stored in liquid nitrogen. Frozen sapphire discs were carefully removed from the aluminum “sandwich” and transferred to 1 mL of pre‐cooled freeze‐substitution medium (0.2% (w/v) osmium tetroxide, 0.1% (w/v) uranyl acetate, 5% (v/v) distilled water in acetone). The samples were placed in a freeze‐substitution unit (AFS2, Leica) and slowly warmed to 0 °C over a 20‐h period. After warming, the freeze‐substituted samples were brought to room temperature, washed three times with acetone at 30‐min intervals, and then infiltrated sequentially with epoxy resin‐acetone mixtures (1:1, 1:2, and 2:1) for 1 h each. The samples were subsequently infiltrated with 100% epoxy resin overnight. Finally, each sample was transferred to a fresh Eppendorf tube containing epoxy resin and polymerized at 60 °C for 24 h. After polymerization, the sapphire discs were detached using liquid nitrogen. The resin blocks, now imprinted with cells, were trimmed and sectioned into 100 nm/80 nm sections using a 45° diamond knife (Diatome) on an EM UC6 ultramicrotome (Leica).

### Temperature Sensing of FND‐CR Using Optically Detected Magnetic Resonance (ODMR)

The temperature sensing on FND‐CR particles was performed using the home‐built confocal fluorescence microscope (details in supplementary information) based on Qudi.^[^ ^63^ ^]^ 10 µL of 10 µg mL^−1^ FND‐CR or FND‐NG was dropped on the oxygen plasma‐cleaned, antenna‐pre‐fabricated coverslip and let dry at room temperature. The continuous‐wave optically detected magnetic resonance (CW‐ODMR) spectrum of isolated nanodiamonds (selected based on photon counts) was first acquired without IR irradiation for ≈5 min. To measure the temperature change of FND‐NG and FND‐CR under IR irradiation (810 nm, 0.6 W cm^−2^, Thorlabs), the IR lamp and the ODMR measurement were synchronously started. The ODMR data was continuously acquired every 5 to 20 min. During post‐processing, the ODMR spectrum was fitted with a double Lorentzian function. The shift in the peak frequencies (*D*_0_) of the NVs ODMR spectrum was used to determine the temperature change at a certain irradiation time. A total of seven FND‐CR particles (n = 7) and three FND‐NG particles (n = 3) were analyzed under identical conditions, with FND‐NG serving as a negative control to confirm CR‐mediated photothermal heating.

### Sample Preparation and Intracellular Temperature Measurements Using ODMR

100 µL of 3 × 10^5^ cells mL^−1^ J774A.1 suspension was added to coverslips equipped with the linear gold antenna and 2‐well cell inserts (ibidi). After overnight incubation, 100 µL of 20 µg mL^−1^ FND‐CR or 10 µg mL^−1^ FND‐NG in cell culture medium was added to the cells. Subsequently, the cells were washed with DPBS 3 times to remove the non‐internalized FND‐CR nanoparticles, and supplied with fresh Leibovitz's L‐15 Medium. The ODMR measurements were performed immediately. After selecting the cells containing FND‐CR with photon counts ranging from 0.3 to 1 M counts/s, CW‐ODMR was performed following the same procedure as described above. One of the major challenges of the presented intracellular time‐resolved temperature sensing experiment was the sudden positional drift of the FND‐CR nanoparticles. Such positional drift can be corrected by using real‐time particle tracking techniques. Although full‐spectrum ODMR provides slower acquisition compared to accelerated methods, it offers distinct advantages for accurate temperature readout during CR‐induced heating. Faster acquisition can be achieved by monitoring fluorescence intensity at a temperature‐sensitive point of the ODMR spectrum, near the steepest slope of the resonance line, where changes in zero‐field splitting (*D*_0_) induce maximal fluorescence variation for a given temperature shift.^[^ ^64^ ^]^ Alternatively, accelerated measurements can be performed using a four‐point sampling method, which probes selected microwave frequencies on both sides of the resonance to approximate *D*_0_ shifts with reduced acquisition time by Kucsko et al.,^[^ ^19^ ^]^ However, both approaches are highly sensitive to ODMR linewidth variations, which can fluctuate due to intracellular microenvironments and heating. Full‐spectrum ODMR fitting, by contrast, enables robust extraction of *D_0_* values even under changing linewidth conditions, ensuring greater accuracy and reliability in complex intracellular measurements. A total of 6 FND‐CR particles and 3 FND‐NG particles (negative control) were analyzed under identical conditions.

### Sample Preparation and Intracellular Radical Sensing Using Diamond Relaxometry

100 µL of 3 × 10^5^ cells/mL J774A.1 suspension was added to coverslips equipped with the linear gold antenna and 2‐well cell inserts (ibidi) one day before. After incubating macrophages with 30 µg mL^−1^ FND‐CR or 10 µg mL^−1^ FND‐NG overnight, the medium was replaced with fresh Leibovitz's L‐15 Medium. For NAC‐treated groups, cells were preincubated with 10 mm N‐acetyl‐L‐cysteine (NAC) for 30 min, followed by maintenance in medium containing 5 mM NAC during NIR irradiation and relaxometry measurement. First, a fluorescence scan was performed in a small range of 200 µm × 200 µm on the *X–Y* plane as described in the last section using the home‐built confocal microscopy. *T*_1_ measurements were initiated on selected diamond particles for 10 min while continuous particle tracking and autofocus were maintained using a tracking algorithm inspired by Feng et al.^[^ ^65^ ^]^ Following this, the cells were irradiated with an NIR lamp (810 nm, 0.6 W cm^−^^2^) for 15 min. During irradiation, *T*_1_ measurements were temporarily paused, but particle tracking remained active to ensure measurements resumed on the same FND particles. After irradiation, *T*_1_ measurements were resumed and recorded over 60 min, with data collected at 10, 15, 20, 30, 40, 50, and 60 min post‐irradiation. *T*_1_ relaxation times were extracted by fitting the fluorescence decay as a function of the waiting time (τ) using a single‐exponential model. The post‐irradiation *T*_1_ values were normalized to the pre‐irradiation *T*_1_ values to assess relative radical load changes compared to non‐irradiated. Data were reported as mean ± standard deviation (n = 4) for FND‐CR‐treated cells, with FND‐NG‐treated cells serving as a negative control.

### Macrophage Polarization Assay

3 × 10^4^ cells/well of J774A.1 were seeded into a 24‐well plate one day before. Then the cells were incubated with 30 µg mL^−1^ FND‐CR overnight. Then, cells were irradiated under a 810 nm NIR lamp for 20 min at different laser intensities: 0, 0.3 W cm^−2^ (½ IR), or 0.6 W cm^−2^ (IR), following removal of the non‐internalized FND‐CR by replacing the cells with L‐15 medium, namely, FND‐CR, FND‐CR + ½ IR, and FND‐CR + IR, respectively. Each experimental condition was performed in two independent replicates to ensure reproducibility. The effect of NIR irradiation was also investigated by irradiating the cells without FND‐CR incubation at 0.6 W cm^−2^ for 20 min. After irradiation, the L‐15 medium was replaced with normal DMEM medium and incubated for another 24 h. The cells without any treatment were set as the negative control group, and the cells treated with 1X LPS (ThermoFisher Science, Germany) for 20 h were set as the positive control. For NAC‐treated groups, cells were preincubated with 10 mm NAC for 30 min, followed by maintenance in medium containing 5 mm NAC during NIR irradiation or co‐incubation with 1X LPS. Afterward, cells with different treatments were incubated with 5 µg mL^−1^ CD16/32 antibody (Cat: 14‐0161‐82, ThermoFisher Science) in staining buffer (1% BSA and 0.09% sodium azide in DPBS) for 15 min at 4 °C following being washed by DPBS twice, harvested gently by cell scrapers, and collected after centrifuging at 400 xg for 5 min at 4 °C. Next, the cells were stained with 0.6 µg mL^−1^ CD80 monoclonal antibody‐PE (anti‐CD80‐PE, Cat: 12‐0801‐82, ThermoFisher Science) or 1.25 µg mL^−1^ CD86 monoclonal antibody‐PE (anti‐CD86‐PE, Cat: 12‐0862‐82, ThermoFisher Science) for 30 mins at 4 °C, followed by washing with DPBS twice and analyzed by a flow cytometer (Novocyte Quanteon, Agilent). At least 10 000 events were analyzed. The flow cytometry data were analyzed by FlowJo. In addition, stained cells without harvest were also imaged by a wide field microscopy (BZX800, Keyence) using a 20x air objective (N.A. 0.4). The PE signal was imaged using the TIRTC filter.

### Transcriptome Assay

5 × 10^4^ cells/well were seeded and treated with the same procedure as described above. Each experimental condition was performed in three independent replicates to ensure reproducibility. Then the cells were washed with DPBS twice and harvested. Cell pellets were lysed and RNA was extracted using the RNeasy Plus Micro Kit (Qiagen) according to the supplier's instructions. RNA quantity was measured using the Qubit Fluorometer (Thermo Fisher), and RNA quality was measured using the Tape Station (Agilent). Samples with insufficient RNA quantity were omitted from downstream transcriptome analysis. Library preparation and sequencing (30mio reads/sample; PE150) were performed by Novogene. Data processing and analysis was performed using the Qiagen CLC Genomics Workbench (v24.0.1) and the following settings: Reference sequence = Mus musculus (GRCm39); Gene track = Mus musculus (GRCm39.111); mRNA track = Mus musculus (GRCm39.111); Use spike‐in controls = no; Mismatch cost = 2; Insertion cost = 3; Deletion cost = 3; Length fraction = 0,8; Similarity fraction = 0,8; Strand specific = Both; Library type = Bulk; Ignore broken pairs = Yes; Expression value = TPM. Heatmaps were created using Morpheus software (Morpheus). Comparative pathway enrichment analysis was performed by DAVID.^[^ ^66^, ^67^ ^]^

## Conflict of Interest

The authors declare no conflict of interest.

## Author Contributions

Y.W. and T.W. conceived the idea and supervised the projects. K.W., Y.W., and T.W. conceptualized and designed the experiments. Y.R. established the synthesis of the photothermal molecular, K.W. synthesized the photothermal molecular and nanodiamond hybrid. K.W. and Q.L. performed the confocal and TEM imaging. P.B. and F.J. assisted the upgrade of the ODMR microscope and modified the operation software for data acquisition. K.W. performed the ODMR measurements, flow cytometry experiments, and data analysis. T.B. and T.B. conceptualized transcriptome experiments and interpreted the transcriptome results. K.E.‐J., H.K., and M.K. performed the transcriptome experiment and the transcriptome analysis, and, K.E.‐J. and H.K., performed the primary cell experiments under the supervision of T.B., T.B., and K.W. wrote the first draft of the manuscript with input from all authors, and all authors contributed to the manuscript revision.

## Supporting information



Supporting Information

## Data Availability

The data that support the findings of this study are available from the corresponding author upon reasonable request.
