# Supplementary material for: Single‐Cell Hyperthermia: Diamond Quantum Thermometry Reveals Thermal Control of Macrophage Polarization
Source: Adv Mater. 2025 Dec 7;38(8):e17076. doi: 10.1002/adma.202517076 (PMC12878808; doi:10.1002/adma.202517076)
Supplement: Supplementary file 1 — Supporting Information [file ADMA-38-e17076-s001.docx]

Supplementary Information for

**Single-Cell Hyperthermia: Diamond Quantum Thermometry Reveals Thermal Control of Macrophage Polarization**

Kaiqi Wu^1^, Qi Lu^1^, Yong Ren^1^, Priyadharshini Balasubramanian^2^, Kazem Ebadi Jalal^3^, Hannah Klug^3^, Matthias Klein^3,4^, Toszka Bohn^3,4,5^, Tobias Bopp^3,4,5,6,7,8^, Fedor Jelezko^2^, Yingke Wu^1^*, Tanja Weil^1^*

^1^Max Planck Institute for Polymer Research, Ackermannweg 10, 55128 Mainz, Germany

*Corresponding author. E-mail: weil@mpip-mainz.mpg.de, wuyingke@mpip-mainz.mpg.de.

^2^Institute for Quantum Optics, Ulm University, Albert-Einstein-Allee 11, 89081 Ulm, Germany

^3^Institute of Immunology, University Medical Center of the Johannes Gutenberg University Mainz, Langenbeckstraße 1, 55131 Mainz, Germany

^4^Research Center for Immunotherapy (FZI), University Medical Center Mainz, 55131 Mainz, Germany

^5^German Cancer Consortium (DKTK), 69120 Heidelberg, Germany

^6^University Cancer Center (UCT) Mainz, University Medical Center Mainz, 55131 Mainz, Germany

^7^Institute for Quantitative and Computational Biosciences (IQCB), 55128 Mainz, Germany

^8^Centre for Healthy Ageing, Johannes Gutenberg-University Mainz, 55128 Mainz, Germany

**Table of Contents**

[Materials 4](#_Toc213541492)

[Synthetic Strategy of Photothermal Dye 4](#_Toc213541493)

[Photothermal Profile Study for pure CR and FND-CR in Water Using Thermal Camera 5](#_Toc213541494)

[Fabrication of a Linear Gold Antenna on the Coverslips 5](#_Toc213541495)

[Home-built Confocal Microscopy for Optically Detected Magnetic Resonance 6](#_Toc213541496)

[Diamond Relaxometry 6](#_Toc213541497)

[Cytotoxicity Assays of CR, CR-PEG, and FND-CR 7](#_Toc213541498)

[Early Apoptosis Detection with Annexin V staining 7](#_Toc213541499)

[General intracellular ROS detection using DCFH-DA 8](#_Toc213541500)

[Experiments on Primary BMDMs 8](#_Toc213541501)

[Supplementary Figures 10](#_Toc213541502)

[Table S1. Comparison of representative biomedical thermometry methods 10](#_Toc213541503)

[Figure S1. Synthetic route of the croconium Dye 11](#_Toc213541504)

[Figure S2. Preparation of FND-CR 11](#_Toc213541505)

[Figure S3. ^1^H NMR spectrum of Compound 1 12](#_Toc213541506)

[Figure S4. ^1^H NMR spectrum of Compound 2. 12](#_Toc213541507)

[Figure S5. ^1^H NMR spectrum of CR (Compound 3). 13](#_Toc213541508)

[Figure S6. LC-MS study of compound CR (Compound 3). 13](#_Toc213541509)

[Figure S7. ^1^H NMR spectrum of compound mPEG-CR 14](#_Toc213541510)

[Figure S8. LC-MS study of compound mPEG-CR. 14](#_Toc213541511)

[Table S2. Size and ζ-potential of FND, FND-NG, and FND-CR 15](#_Toc213541512)

[Figure S9. Loading efficiency and spectrum of FND-CR. 15](#_Toc213541513)

[Figure S10. Photothermal heating profile of CR and FND-CR 15](#_Toc213541514)

[Figure S11. CLSM images of FND-CR in J774A.1 macrophage 16](#_Toc213541515)

[Figure S12. Homebuilt ODMR setup for measuring thermal profile of FND-CR. 17](#_Toc213541516)

[Figure S13. Diamond relaxometry detects paramagnetic noise 17](#_Toc213541517)

[Figure S14. Intracellular ROS levels detected by DCFH-DA 18](#_Toc213541518)

[Figure S15. Cytotoxicity of CR, CR-PEG, and FND-CR in J774A.1 macrophage 20](#_Toc213541519)

[Figure S16. Early apoptosis and cell necrosis assays 20](#_Toc213541520)

[Figure S17.  Transcriptome heatmap showing expression levels of the HSP family. 22](#_Toc213541521)

[Table S3. Functions of Genes listed in Figure 4B in the Manuscript. 22](#_Toc213541522)

[Figure S18. Lysosomal membrane rupture appeared after Irradiation of FND-CR. 23](#_Toc213541524)

[Figure S19. TNF pathway enrichment assays. 25](#_Toc213541525)

[Figure S20. Representative fluorescence images of anti-CD80/86-PE stained J774A.1 26](#_Toc213541526)

[Figure S21. Gating example of flow cytometry analysis. 27](#_Toc213541527)

[Figure S22. Flow cytometry analysis of J774A.1 polarization 28](#_Toc213541528)

[Figure S23. Intracellular oxidative stress assay using NAC as a ROS scavenger. 29](#_Toc213541529)

[Figure S24. Flow cytometry analysis of NAC-treated J774A.1 cells. 30](#_Toc213541530)

[Figure S25. NAC-mediated ROS scavenging in LPS-stimulated J774A.1 macrophages. 31](#_Toc213541531)

[Figure S26. Flow cytometry analysis of BMDMs 32](#_Toc213541532)

[Figure S27. Intracellular temperature change readout in J774A.1 and BMDMS 33](#_Toc213541533)

[References 33](#_Toc213541534)

# Materials

Nanodiamonds containing nitrogen-vacancy (NV) centers, with an average hydrodynamic diameter of 40 nm, were obtained from Adamas Nanotechnologies. Four-arm PEG-succinimidyl carboxymethyl ester (PEG-SCM, MW: 10 kDa) was obtained from Creative PEG Works. Branched polyethylenimine (PEI, MW: 25 kDa by light scattering), polyvinylpyrrolidone (PVP, MW: 10 kDa), 2-thiophene thiol, croconic acid, hexafluorophosphate azabenzotriazole tetramethyl uranium (HATU), N,N-di*iso*propylethylamine (DIEA), 1-ethyl-3-(3-dimethylaminopropyl)carbodiimide hydrochloride (EDCI), N-hydroxysuccinimide (NHS), and poly(ethylene glycol) methyl ether amine (mPEG-NH₂) were purchased from Sigma-Aldrich. Methyl 4-piperidine-carboxylate was acquired from Tokyo Chemical Industry Co., Ltd. (TCI). All solvents and chemicals, including toluene, ethyl acetate, n-hexane, n-butanol, acetonitrile, and N, N-dimethylformamide (DMF), were purchased from commercial suppliers and used as received, without additional purification.

Cell line and biological reagents. The J774A.1 mouse macrophage cell line (ACC 170) was purchased from the Leibniz Institute DSMZ–German Collection of Microorganisms and Cell Cultures GmbH. Dulbecco's Modified Eagle Medium (DMEM, 1x), Leibovitz's L-15 Medium, Dulbecco's Phosphate-Buffered Saline (DPBS, 1x), Fetal Bovine Serum (FBS), and Penicillin-Streptomycin (Pen Strep) were provided by Gibco, Thermo Fisher Scientific. The antibodies anti-CD80-PE (catalog number: 12-0801-82), anti-CD86-PE (catalog number: 12-0862-81), and anti-CD16/32 (catalog number: 14-0161-82) were purchased from eBioscience, Thermo Fisher Scientific. The CellTiter-Glo Luminescent Cell Viability Assay kit was obtained from Promega.

# Synthetic Strategy of Photothermal Dye

**Synthesis of Croconium Dye.** The croconium dye was synthesized following a previously reported method (reference 16 in the Main text). The synthetic route is illustrated in Figure S1. Briefly, 639.1 mg (5.5 mmol) of 2-thiophenethiol and 945.2 mg (6.6 mmol) of methyl 4-piperidinecarboxylate were combined in 15 mL of toluene and refluxed for 2 hours. After cooling to room temperature, the reaction mixture was filtered and washed with 100 mL of ethyl acetate. The solvents from the combined filtrates were removed under reduced pressure, yielding a light yellow solid. The crude product was purified via column chromatography using silica gel as the stationary phase and a 5:1 (v/v) mixture of hexane and ethyl acetate as the eluent. The purified compound, methyl 1-(thiophene-2-yl)piperidine-4-carboxylate (**compound 1**), was obtained as a light yellow solid with a yield of 64%.

^1^H NMR (400 MHz, acetone-*d*_6_): δ 6.74 (dd, *J* = 5.4, 3.7 Hz, 1H), 6.64 (dd, *J* = 5.5, 1.4 Hz, 1H), 6.15 (dd, *J* = 3.8, 1.4 Hz, 1H), 3.66 (s, 3H), 3.53 – 3.43 (m, 2H), 2.88 – 2.76 (m, 2H), 2.49 (tt, *J =* 11.1, 3.9 Hz, 1H), 2.03 – 1.94 (m, 2H), 1.86 – 1.73 (m, 2H). ESI: *m/z* calculated 225.3, found 226.3 as [M + H]^+^.

Next, 755.0 mg (3.4 mmol) of **compound 1** was dissolved in 15 mL of 0.5 M sodium hydroxide solution and refluxed for 1 hour. After cooling, the pH of the reaction mixture was adjusted by the gradual addition of 10% acetic acid, resulting in the formation of a precipitate. The precipitate was collected by filtration, and the product was dried under vacuum to afford 1-(thiophene-2-yl)piperidine-4-carboxylic acid (**compound 2**) as a light blue powder, with a yield of 78%.

^1^H NMR (400 MHz, MeOD): δ 6.73 (dd, *J* = 5.5, 3.8 Hz, 1H), 6.64 (dd, *J* = 5.5, 1.4 Hz, 1H), 6.18 (dd, *J* = 3.8, 1.4 Hz, 1H), 3.49 (dt, *J* = 11.9, 3.6 Hz, 2H), 2.81 (td, *J* = 12.2, 2.9 Hz, 2H), 2.42 (tt, *J* = 11.1, 3.9 Hz, 1H), 2.04 – 1.97 (m, 2H), 1.89 – 1.77 (m, 2H). ESI: *m/z* calculated 211.1, found 212.1 as [M + H]^+^.

Subsequently, 422.1 mg (2.0 mmol) of **compound 2** and 142.2 mg (1.0 mmol) of croconic acid were dissolved in a mixture of toluene and n-butanol (20 mL, 1:1 v/v). The reaction was stirred under reflux for 1 hour. After cooling to room temperature, the mixture was filtered and washed with methanol. The product was then dried under vacuum to yield pure croconium dye (**compound 3**) as a black powder, with a 76% yield. ^1^H NMR (400 MHz, DMSO-*d*_6_): δ 12.34 (s, 2H), 8.51 (s, 2H), 7.03 (d, *J* = 5.1 Hz, 2H), 3.99 (d, *J* = 13.6 Hz, 4H), 3.52 (t, *J* = 11.2 Hz, 4H), 2.72 – 2.61 (m, 2H), 2.04 (dd, *J* = 13.8, 4.0 Hz, 4H), 1.80 – 1.66 (m, 4H). ESI: *m/z* calculated 528.1, found 528.1 as [M]^+^ and 551.1 as [M + Na]^+^.

**Synthesis of Methoxy Polyethylene Glycol-Croconium (mPEG-CR).** To a solution of 52.8 mg croconium dye (**CR**, **Compound 3**, 0.1 mmol), 38.3 mg poly(ethylene glycol) methyl ether amine (mPEG-NH₂, MW: 383.5 g/mol, 0.1 mmol), and 65.0 mg N, N-di*iso*propylethylamine (DIEA, 0.5 mmol) in 5 mL dimethylformamide (DMF), 38.0 mg hexafluorophosphate azabenzotriazole tetramethyl uronium (HATU) were added. The reaction mixture was stirred at room temperature overnight. After solvent removal using a rotary evaporator, the crude product was purified by preparative high-performance liquid chromatography (Prep-HPLC, Shimadzu Nexera) with acetonitrile (ACN) and water as the mobile phase. The desired mPEG-CR was obtained as a black solid in 39% yield.

^1^H NMR (400 MHz, DMSO-*d*_6_): δ 12.22 (s, 1H), 8.53 (s, 2H), 7.98 (t, *J* = 5.7 Hz, 1H), 7.05 (q, *J* = 4.9 Hz, 2H), 4.04 (dd, *J* = 27.1, 13.6 Hz, 4H), 3.58 – 3.47 (m, 28H), 3.44 – 3.40 (m, 4H), 3.24 (s, 3H), 3.22 (d, *J* = 5.9 Hz, 2H), 2.71 – 2.65 (m, 1H), 2.57 – 2.54 (m, 1H), 2.05 (dd, *J* = 13.6, 3.3 Hz, 2H), 1.96 – 1.86 (m, 2H), 1.74 (q, *J* = 11.4 Hz, 4H). ESI: *m/z* calculated 893.3, found 894.4 as [M + H]^+^ = 894.4 and 916.4 as [M + Na]^+^.

# **Photothermal Profile Study for pure CR and FND-CR in Water Using Thermal Camera**

To investigate the photothermal temperature changes under near-infrared (NIR) irradiation, 100 µL of dissolved CR and dispersed FND-CR, all prepared with the same CR concentration (0.1 mM), were exposed to NIR irradiation (810 nm, 0.6 W/cm², Thorlabs). A thermal camera (Testo 880) was used to capture images and record the temperature of the solutions at 1-minute time intervals throughout the irradiation period.

# Fabrication of a Linear Gold Antenna on the Coverslips

The linear gold antenna was designed and fabricated on coverslips using a lithography process, as described by Oshimi et al.(reference 30 in the Main text). Briefly, pre-cleaned coverslips were used as substrates for coating with S1813 photoresist. The coverslips were exposed to UV light for 40 seconds through a designed mask, followed by development in MF-26 developer. After rinsing with water, a 40 nm gold film was sputtered onto the coverslips. Finally, the coverslips were rinsed with acetone and water, then dried with nitrogen gas. These coverslips were subsequently used for seeding nanodiamond particles, J774A.1 cells, and for optically detected magnetic resonance (ODMR) measurements.

# Home-built Confocal Microscopy for Optically Detected Magnetic Resonance

Temperature sensing was performed using a home-built optically confocal microscope, as previously described (Reference 19 in the Main text). Briefly, a 532 nm continuous-wave laser (Laser Quantum, gem 532) was used to excite the NV centers within the nanodiamonds. The laser beam was first directed onto a TeO₂ acousto-optic modulator (AOM) (Crystal Technology 3200-146), which functioned as an optical switch for pulsed experiments. Following the AOM, the beam was coupled into a single-mode fiber, which acted as a mode cleaner by allowing only the fundamental Gaussian TEM₀₀ mode to pass through. This step was essential to correct any mode distortion caused by the AOM. The outcoupled laser light then passed through a 530 nm notch filter (Chroma Technology HQ530/30M) to remove any residual fluorescence originating from the fiber. A λ/2 waveplate (Thorlabs WPH10M-532) was used for polarization adjustment of the laser beam. The beam was then directed by a mirror to a beam sampler (BS) (Thorlabs BSF20-B), which primarily transmits the NV center fluorescence while reflecting a portion of the green excitation light toward the sample.

A 100× oil-immersion objective (Nikon PLAN 100x, N.A. = 1.35) was employed to focus the green laser onto the diamond sample and to collect the emitted fluorescence. Sample positioning was achieved using a 3D piezostage (Piezoconcept LT3) with a scanning range of 200 × 200 × 100 μm and a positioning accuracy of 0.2 nm. The collected fluorescence passed back through the beam sampler and was subsequently focused onto a 25 μm pinhole (Thorlabs PS25) to eliminate out-of-focus light, enabling optical sectioning and high-resolution imaging from both the surface and interior of the sample. The fluorescence was filtered with a 640/75 nm band pass filter and detected using an avalanche photodiode (APD) (Laser Components, COUNT-100C) for microwave (MW) delivery, a signal generator (Rohde & Schwarz SMIQ03B) along with a microwave amplifier (Mini-Circuits ZHL-15W-422-S+) was used to achieve the required power levels for ODMR measurements. The entire experiment was controlled by the Qudi open-source software package (Reference 63 in the Main text), specifically customized for this setup.

# Diamond Relaxometry

The longitudinal spin relaxation time (*T*_1_) of the NV centers in the FND-CR was measured using afore described home-built confocal microscope. The NV centers were excited using a 532 nm laser. The pulse sequence consists of a series of 10 μs long laser pulses (Supplementary Fig. 13b). The laser pulse polarizes the NV centers to the | *m*_s_ = 0 ⟩ spin state. After a variable waiting time (τ), the subsequent laser pulse reads out the spin state of the NV centers. The fluorescence photons detected in the laser pulse's first 300–500 ns contain the spin state information and hence constitute the signal. The *T*_1_ measurement data shown was normalized, i.e., the signal (fluorescence obtained during the first 300 ns) was divided by the reference steady-state fluorescence (fluorescence obtained when the NV center is re-initialized into the | *m*_s_ = 0 ⟩ spin state). The *T*_1_ measurement sequence was repeated several times with a total acquisition time of 15 minutes. The measured fluorescence data were plotted as a function of the waiting time (τ) between the laser pulses and fitted with a mono-exponential function of the form I_0_+A exp(-τ /*T*_1_).

To verify the paramagnetic sensing capability of the NV centers after modification, FND-CR samples were prepared by depositing 20 μL of a 10 μg/mL FND-CR suspension onto an O₂-plasma-cleaned 18-well μ-slide (ibidi). The samples were dried overnight before measurement. *T*_1_ relaxation measurements were conducted using the home-built confocal microscope described above. Only single FND-CR particles with moderate fluorescence count rates (300,000–1,000,000 counts/s) were selected for analysis. *T*_1_ measurements were performed on the same FND-CR particles in GdCl₃ solution with different concentrations (0 mM, 0.1 mM, and 100 mM). *T*_1_ relaxation times were extracted by fitting the fluorescence signal as a function of the waiting time (τ) using a single-exponential decay model. In total, *T*_1_ measurements were carried out on 10 individual, well-isolated fluorescence spots.

# Cytotoxicity Assays of CR, CR-PEG, and FND-CR

J774A.1 cells were cultured in DMEM medium supplied with 10% (v/v) FBS, 100 U/mL penicillin, and 100 µg/mL streptomycin (termed as complete DMEM medium) at 37 °C in a humidified incubator with 5% CO₂ atmosphere. For cytotoxicity assays, J774A.1 cells were seeded at a density of 2,500 cells per well in white opaque half-area 96-well plates (ThermoFisher) and incubated overnight to allow cell attachment. Triplicate wells were used for each concentration in all conditions. The next day, serial dilutions of test compounds were prepared. CR and CR-PEG were diluted in DPBS to prepare 10 times stock solutions at final concentrations (1, 50, 100, 500, and 1000 µM). FND-CR were similarly diluted to 10× stocks corresponding to final concentrations of 1.25, 12.5, 25, 250 µg/mL, 1.25 mg/mL, and 5 mg/mL. From each 10× stock, 20 µL was added to 180 µL of complete DMEM medium to prepare work solutions. Prior to treatment, culture medium in each well was aspirated and replaced with 50 µL of fresh complete medium containing the indicated concentrations of CR, CR-PEG (0.1, 5, 10, 50, and 100 µM), or FND-CR (0.125, 1.25, 2.5, 25, 125, and 500 µg/mL). Cells were then incubated with the test samples for 24 hours under standard culture conditions (37 °C, 5% CO_2_). After incubation, cell viability was assessed using the CellTiter-Glo® Luminescent Cell Viability Assay. Briefly, 50 µL of CellTiter-Glo® reagent was added to each well and the plate was placed on an orbital shaker at room temperature for 10 minutes. Luminescence was measured using a Tecan plate reader to assess cell viability. Relative cell viability was calculated by normalizing luminescence values to untreated control wells.

# Early Apoptosis Detection with Annexin V Staining

J774A.1 cells were grown in an 18-well cell culture slide (2 × 10^5^ cells/mL, 100 µL in each well). The next day, 30 µg/mL FND-CR dispersed in cell culture medium were added to some of the wells (100 µL each). After 4 hours of incubation, cells were washed with DPBS and then treated with 810 nm light same way as earlier mentioned in live/dead staining of J774A.1 cells. Then again, after 4 hours of incubation, the eBioscience™ Annexin V-FITC apoptosis detection kit was used to stain the cells simultaneously with propidium iodide (PI). For each 200 µL diluted binding buffer, 5 µL of Annexin V-FITC and 5 µL PI were mixed, then 100 µL of staining solution was added to each well, which remained in the dark for 15 minutes at room temperature. After 15 minutes, the staining solution was replaced with binding buffer, and immediately, the microscopy imaging was performed.

# General Intracellular ROS Detection using DCFH-DA

Intracellular reactive oxygen species (ROS) generation was assessed using 2′,7′-dichlorofluorescin diacetate (DCFH-DA, eBioscience™). J774A.1 macrophages were seeded in 8-well chamber slides at a density of 1 × 10⁵ cells/mL (300 µL per well) and cultured overnight. Cells were then incubated with FND-CR (30 µg/mL, 200 µL per well) for 4 h, followed by gentle washing with DPBS to remove excess nanoparticles. NIR irradiation (810 nm, 0.6 W/cm², 15 min) was applied as described previously in the live/dead staining assay. After an additional 2 h incubation, cells were treated with 10 µM DCFH-DA diluted in serum-free DMEM (200 µL per well) for 15 min at 37 °C and 5% CO₂.

Subsequently, cells were washed twice with DPBS and resupplied with phenol-red-free L-15 medium supplemented with 10% FBS and 1% pen/strep before imaging on a fluorescence microscope (Keyence). Untreated cells served as negative controls, while LPS-treated cells (1× LPS) were used as positive controls. To confirm ROS specificity, a subset of cells was preincubated with N-acetyl-L-cysteine (NAC, 10 mM, 30 min) prior to treatment with FND-CR, FND-CR + IR, or LPS.

# Experiments on Primary BMDMs

Primary bone marrow–derived macrophages (BMDMs) were prepared following standard differentiation protocols. Briefly, bone marrow cells were harvested from the femurs and tibias of C57BL/6J mice under sterile conditions. Bone marrow plugs were flushed with MEM, supplemented with 2% fetal calf serum (FCS). Cell suspension was then centrifuged (1700rpm, 10min) and resuspended in Gey’s red blood cell lysis buffer for 2min. Afterwards, MEM was added to the cell suspension to stop lysis and passed through a 40µm cell strainer. Bone-marrow cells were resuspended in IMDM (Gibco 12440) supplemented with 10% FCS, 1mM sodium pyruvate, 0,1% Penicillin/Streptomycin, and 15% of L929-derived M-CSF^[^*^1^*^]^ and plated in 6-well suspension culture plates (Greiner) at 3 x 10^6^ cells per well. Cells were cultured for 7 days at 37 °C and 5% CO_2_. Medium was changed every 3 days.

For phototermal experiments fully differentiated macrophages were harvested by gently scraping and replated in IMDM (Gibco 12440) supplemented with 10% FCS, 1mM sodium pyruvate and 0.1% P/S in 24-well plates or chamber slides (1x10^6^/well for the 24 well plates, n=2 wells/condition; 1 x 10^4^/well in chamber slides) and incubated with FND-CR (50 µg/mL) for 20 h. The medium was then changed, and NIR irradiation was performed using an 810 nm LED light source at 0.6 W/cm² (full intensity) or 0.3 W/cm² (half intensity) for 20 min. Untreated cells served as the negative control group. BMDMs were treated either with 20 ng/ml IFN-γ (R&D Systems) and 100 ng/ml Lipopolysaccharide (Sigma) as pro-inflammatory M1-like, or with 20 U/ml IL-4 (in-house production) and 10 ng/ml IL-13 (Peprotech) as anti-inflammatory M2-like macrophages positive control. Cells were then incubated for an additional 48h.

For polarization marker analysis, cells were stained with fixable viability dye eFluor780 (eBioscience), anti-CD80-PE (clone 16-10A1, eBioscience), CD86-BV510 (clone GL-1, Biolegend), CD38-BV605 (clone 90, eBioscience), iNOS-PE-Cy7 (clone CXNFT, eBioscience), CD11b-BV421 (clone M1/70, Biolegend), and F4/80-BV785 (clone BM8, Biolegend) antibodies and analyzed using a BD LSR II device. Flow cytometry data were processed and analyzed with FlowJo (version 10.8.1). For surface staining, cells were stained for 15 min at 4°C with antibodies for the respective cell surface markers. Intracellular staining was performed with a FoxP3 staining kit (eBioscience). Intracellular staining with respective antibodies was done for 30 min at 4°C.

For temperature measurements, ODMR thermometry was performed as described above for J774A.1 macrophages. Individual lysosome-localized FND-CR particles were tracked before and after irradiation to determine the intracellular temperature change (Δ*T*).

# Supplementary Figures

Table S1. Comparison of representative biomedical thermometry methods ^[^*^2-9^*^]^.

| Method | Principle | Spatial Resolution | Temperature Sensitivity | Photostability/ Limitations | Ref |
| --- | --- | --- | --- | --- | --- |
| Infrared (IR) Thermography | Blackbody Emission Imaging | 10–100 µm | 0.1–0.5 °C | Limited to surface; affected by emissivity and scattering | ^[^*^2, 4, 6, 7^*^]^ |
| Fluorescent Dyes | Intensity/Lifetime | 0.5–1 µm | 0.2–1 °C | Prone to photobleaching; sensitive to pH and ionic strength | ^[^*^2, 6, 7^*^]^ |
| Fluorescent Proteins | Protein Conformational Changes | <500 nm | 0.2–1 °C | Bleaching and drift; pH- and expression-dependent | ^[^*^2, 6^*^]^ |
| Quantum Dots | Spectrum shift or Intensity Change | <100 nm | 0.1–0.5 °C | Blinking and bleaching under excitation | ^[^*^6^*^]^ |
| Upconversion Nanoparticles (UCNPs) | Intensity Ratio between Thermally Coupled Levels | ~100 nm | 0.2–0.5 °C | Photostable; requires high-power excitation | ^[^*^6, 9^*^]^ |
| FNDs | (ODMR shift of NV⁻ centers | <50 nm | <0.05 °C | Completely photostable, pH/ion-insensitive | ^[^*^6-8^*^]^ |

Figure S1. Synthetic route of the croconium dye **(Compound 3).**

Figure S2. Preparation of FND-CR based on the pegylated croconium dye CR (mPEG-CR), fluorescent nanodiamonds (FND), 4-arm polyethylene glycol (PEG)-succinimidyl (SCM) carbonate (PEG-SCM), polyethyleneimine (PEI), and polyvinylpyrrolidone (PVP).


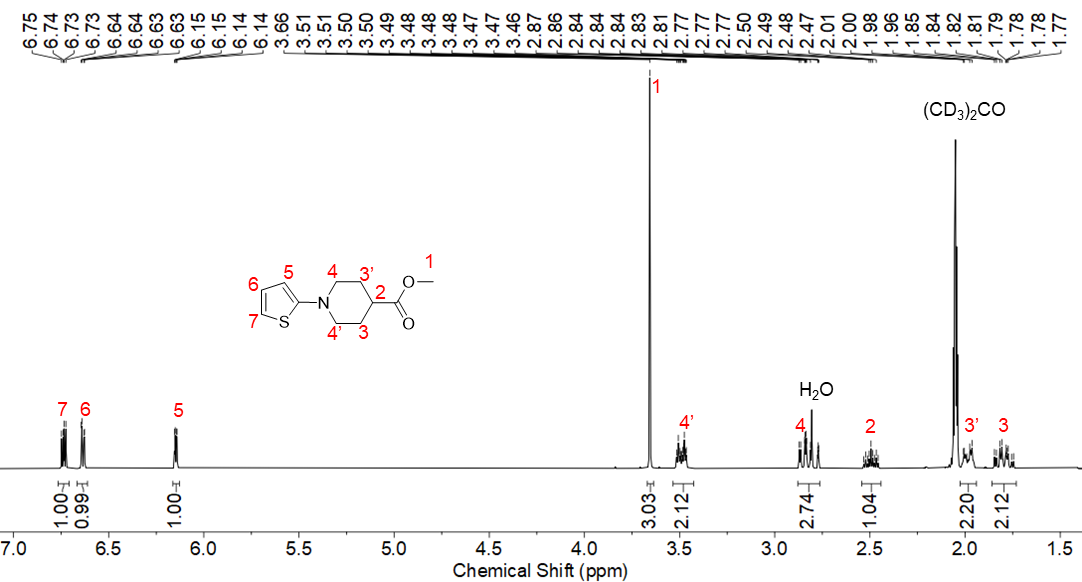


Figure S3. ^1^H NMR (400 MHz, Acetone-*d*_6_) spectrum of Compound 1**.**


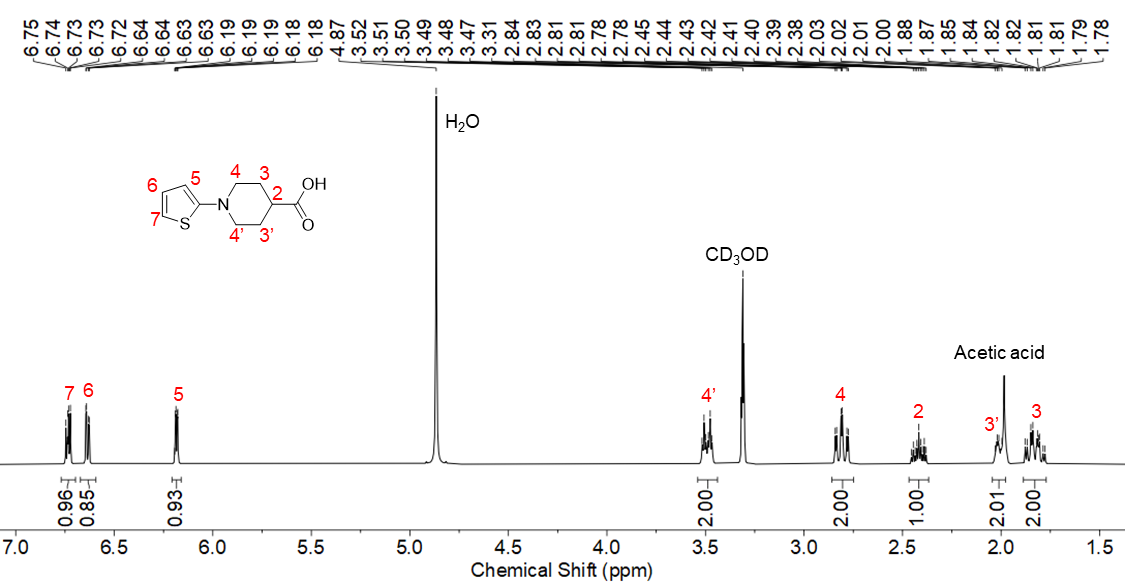


## **Figure S**4. ^1^H NMR (400 MHz, MeOD) spectrum of Compound 2.


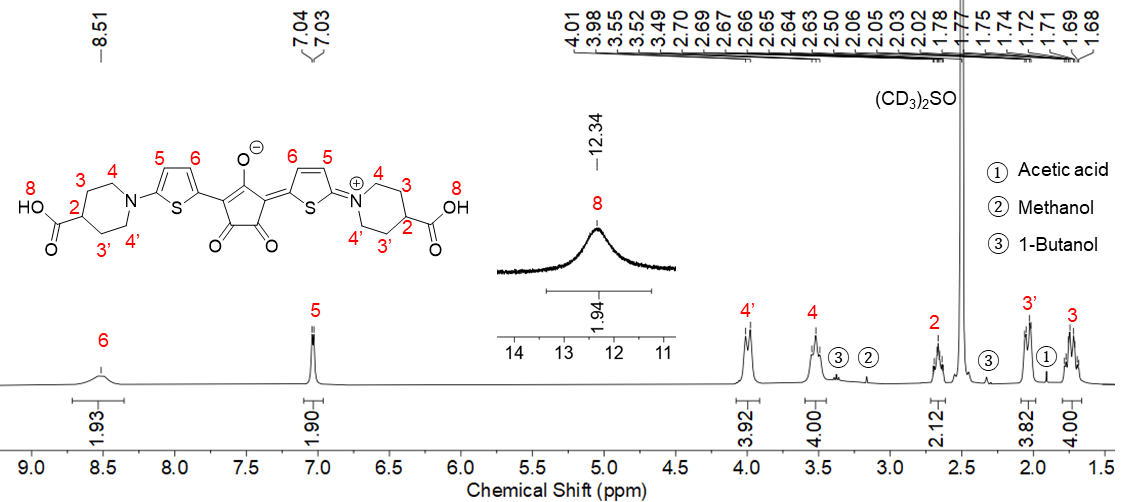


## **Figure S**5. ^1^H NMR (400 MHz, DMSO-*d*_6_) spectrum of CR (Compound 3).

Figure S6. LC-MS study of compound CR (Compound 3). HPLC trace (left) and corresponding MS spectrum (right), showing peaks of [M]^+^ = 528.1 and [M + Na]^+^ = 551.1.

**
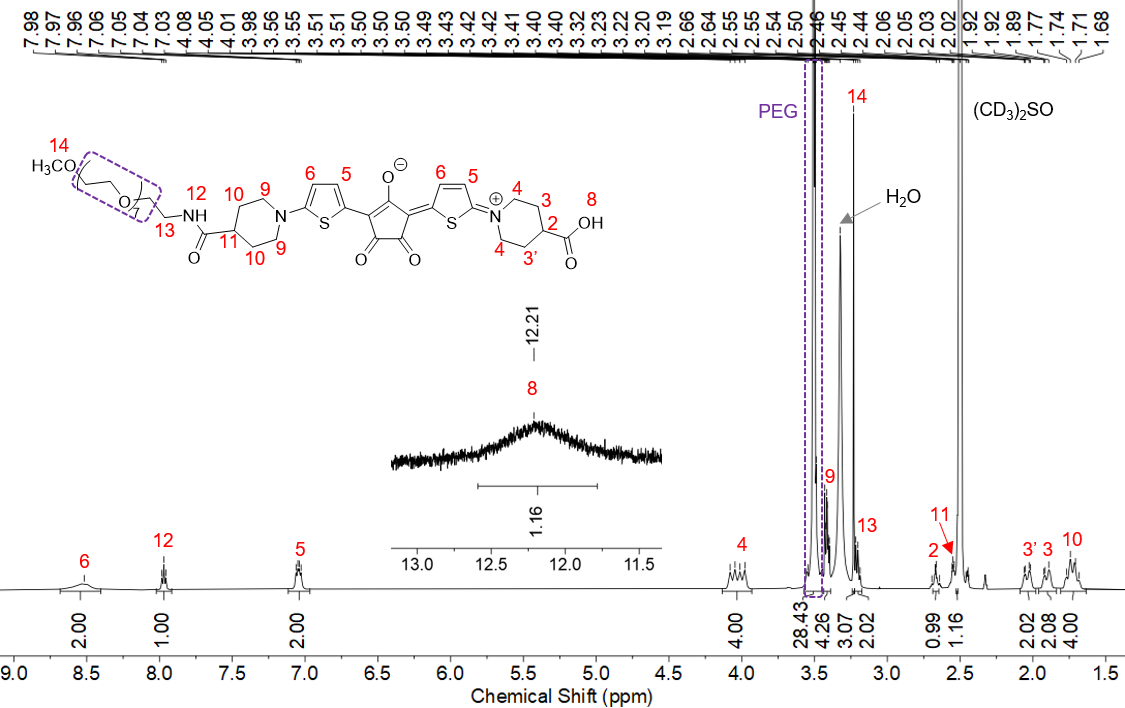
**

Figure S7. ^1^H NMR (400 MHz, DMSO-*d*_6_) spectrum of compound mPEG-CR**.**

Figure S8. LC-MS study of compound mPEG-CR. HPLC trace (left) and corresponding MS spectrum (right), showing peaks of [M + H]^+^ = 894.4 and [M + Na]^+^ = 916.4.

Table S2. Size and ζ-potential of FND, FND-NG, and FND-CR measured by DLS**.**

|  | Size (nm)^a^ | PDI^b^ | ζ-potential (mV) |
| --- | --- | --- | --- |
| FND | 42 ± 2 | 0.13 ± 0.02 | -44.5 ± 2.2 |
| FND-NG | 50 ± 2 | 0.14 ± 0.02 | 25.6 ± 1.5 |
| FND-CR | 55 ± 5 | 0.18 ± 0.01 | -22.4 ± 4.5 |

1. Given by number distribution (n=3).
2. Given by Z-average (n=3).


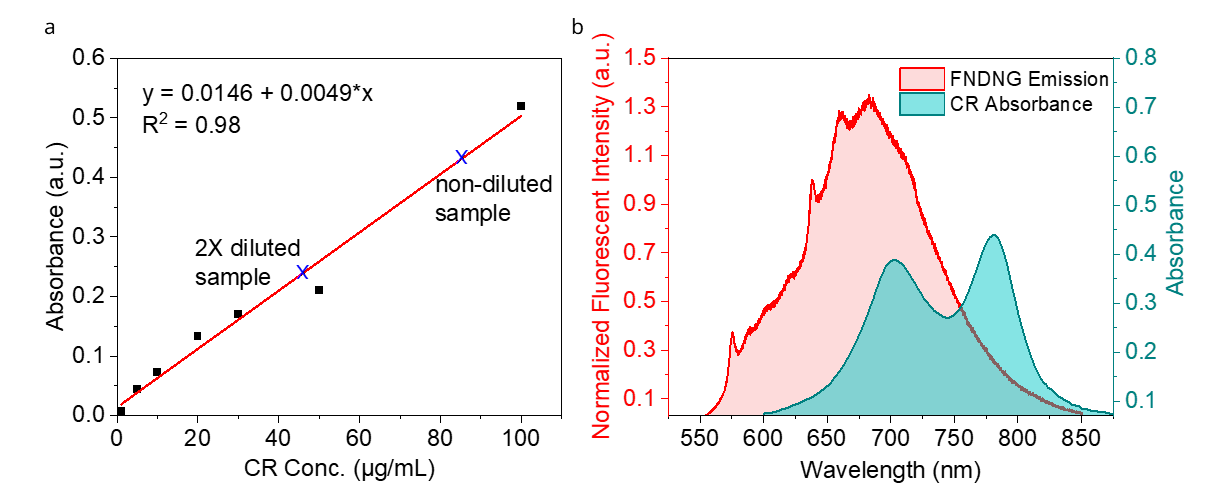


Figure S9. Loading efficiency and spectrum of FND-CR. (**a**) Calibration curve of CR. The blue cross indicates the absorbance of 1 mg/mL and 0.5 mg/mL FND-CR. (**b**) The fluorescent emission spectrum of FND-NG was detected by a home-built confocal microscope, and the NIR absorbance of CR was detected by Tecan.

**
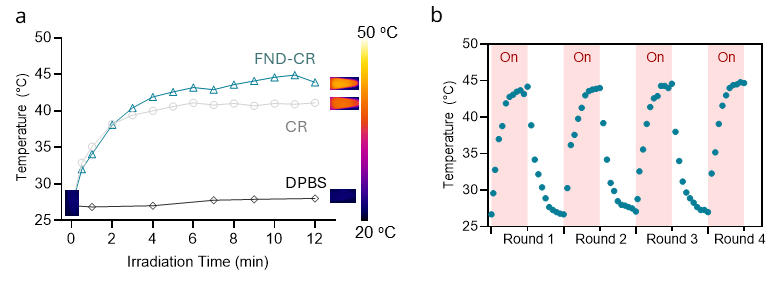
**

Figure S10. (a) Photothermal heating profile of CR and FND-CR at equal CR concentrations (0.1 mM), measured by thermal camera after 12 minutes of NIR irradiation (810 nm, 0.6 W/cm²). (**b**) Thermal cycling of FND-CR measured by a thermal camera, showing no change in heating profile after four cycles.


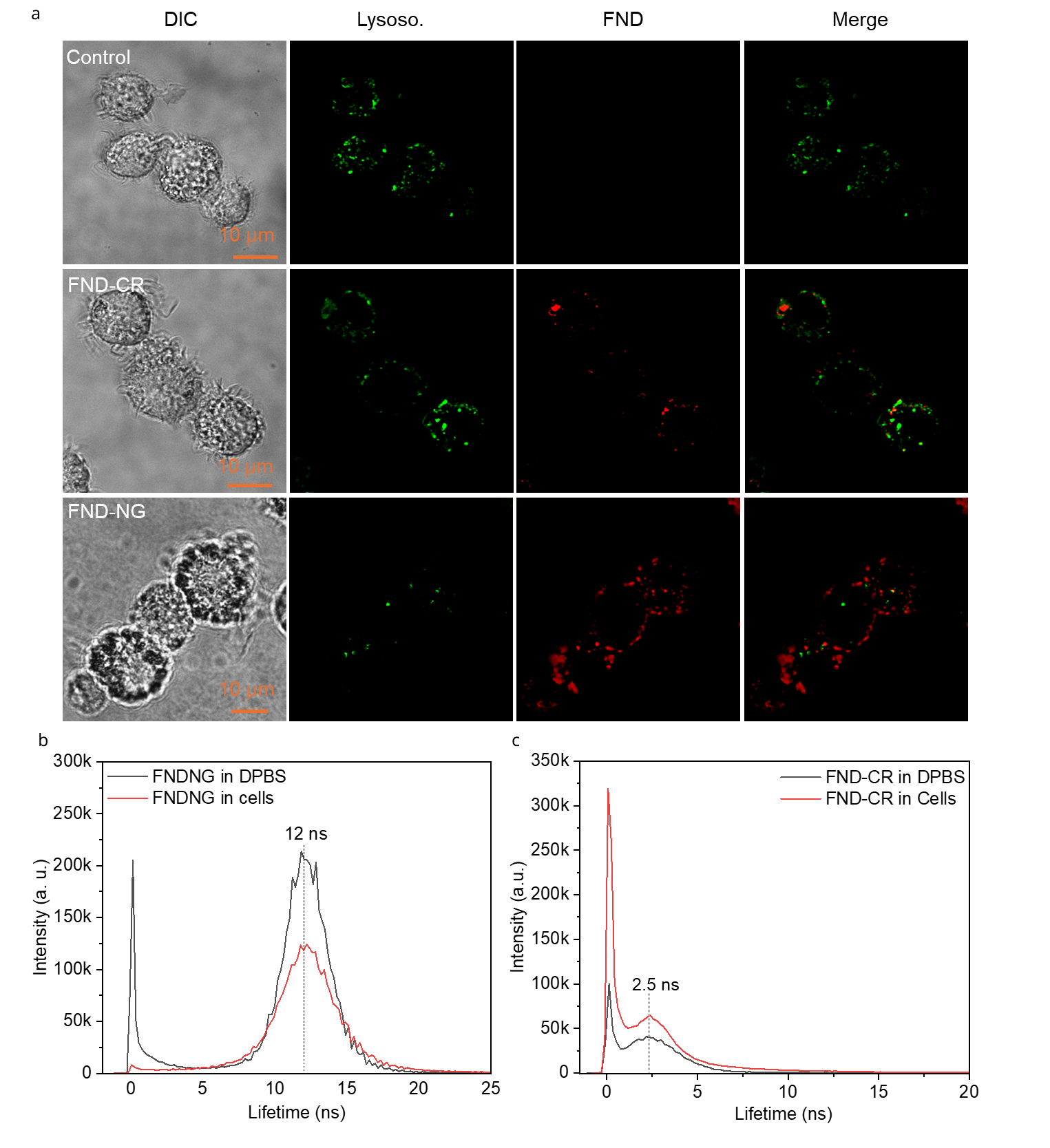


Figure S11. (a) CLSM images of J774A.1 macrophage after incubation with 30 µg/mL FND-CR or 10 µg/mL FND-NG overnight (12 h) and stained with Lysotracker-Green at 37 °C for 30 minutes. (**b** and **c**) Fluorescence lifetime of FND-NG (b) and FND-CR (c) in DPBS and inside the cells was measured by STELLARIS 8 Fluorescence Lifetime Imaging (FLIM). The difference in fluorescence lifetimes between FND-NG and FND-CR may result from the overlap between the emission spectrum of FND-NG and the absorption spectrum of CR.


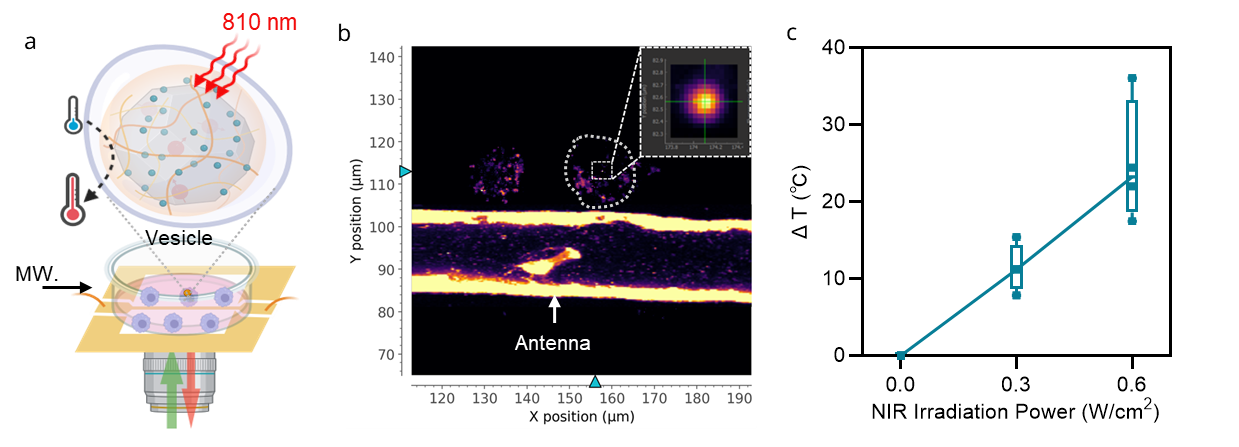


Figure S12. Homebuilt ODMR setup for measuring thermal profile of FND-CR. (**a**) Schematic illustration of the FND-CR location and the corresponding ODMR measurement setup. (**b**) Representative confocal microscopy image captured using a home-built ODMR microscope. The line (Y position range: 85–105 µm) indicates the position of the antenna to load the microwave. The cell boundary is marked with a gray dashed circle. Inset: The selected FND-CR particle used for ODMR measurement. (**c**) Intracellular temperature changes of FND-CR under full power (0.6 W/cm², IR) and half power (0.3 W/cm², ½ IR) of NIR irradiation for 15 minutes (n = 4).


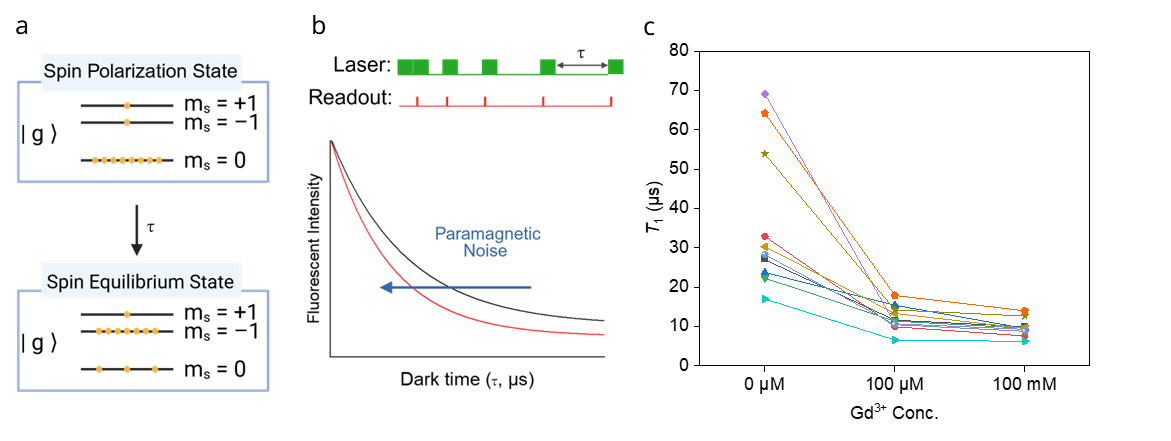


Figure S13. Diamond relaxometry detects paramagnetic noise via spin-state relaxation dynamics. (**a**) Schematic of NV⁻ center spin dynamics. Upon green laser excitation, NV⁻ electrons are initialized into the spin-polarized state (|*m*_s_ = 0⟩). During the dark time (τ), spin relaxation toward equilibrium occurs, which is accelerated by paramagnetic species such as Gd³⁺ or free radicals. (**b**) Pulse sequence and readout strategy. Fluorescence decay is recorded at each τ delay and fitted to extract *T*_₁_ relaxation times. (**c**) *T*₁ relaxation times of individual FND-CR particles measured in response to increasing Gd³⁺ concentrations. Results from 10 particles are shown, demonstrating sensitivity to paramagnetic environments.


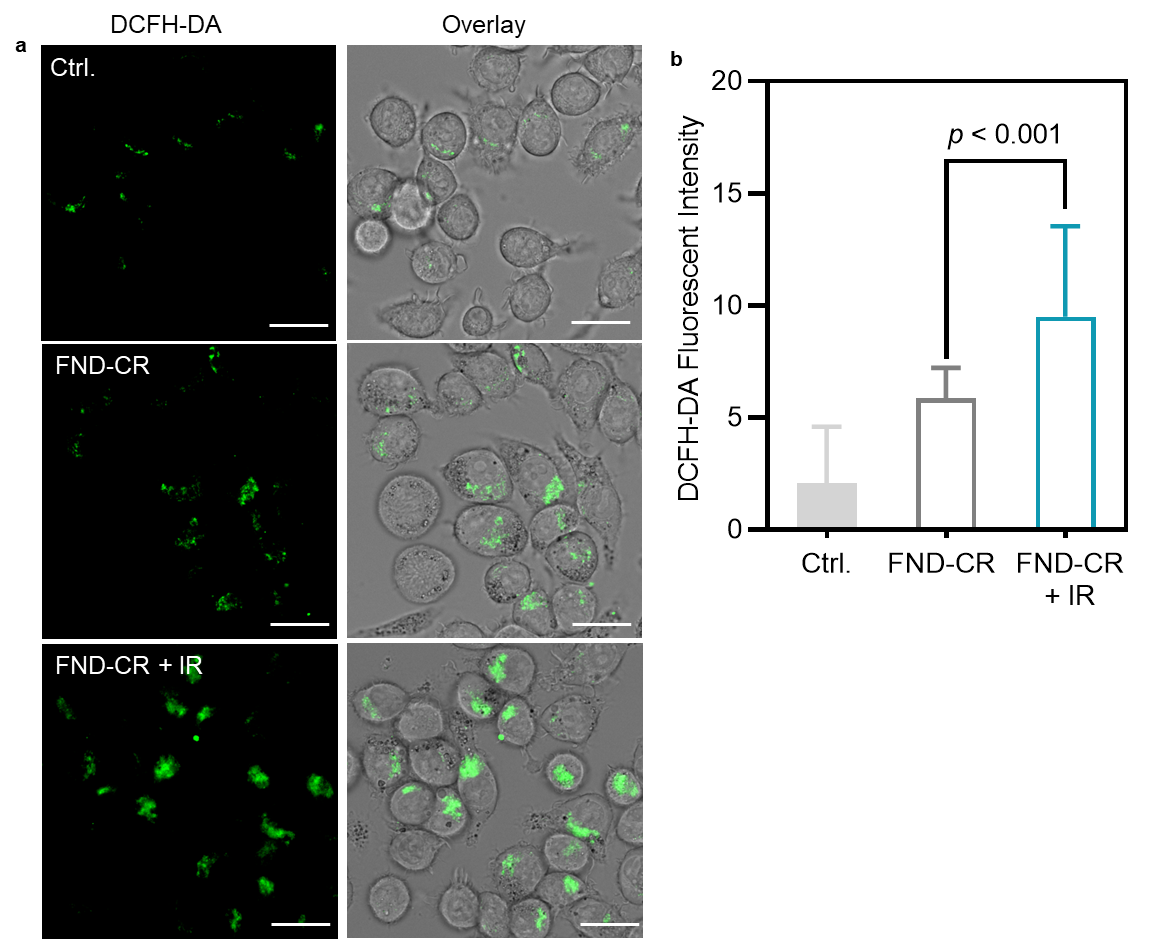


Figure S14. Intracellular reactive oxygen species (ROS) levels in FND-CR and NIR-irradiated J774A.1 macrophages were detected by DCFH-DA staining. (**a**) J774A.1 cells were incubated with FND-CR (30 μg/mL) and irradiated for 15 min (810 nm, 0.6 W/cm²). After irradiation, cells were stained with 10 μM DCFH-DA in serum-free medium for 30 min and imaged using a fluorescence microscope. Scale bar = 20 μm. (**b**) Quantification of DCFH-DA fluorescence intensity from single-cell analysis (n= 50 cells). Data are presented as mean + SD. A two-tailed unpaired *t-test* was used to calculate statistical significance, with *p*-values indicated.


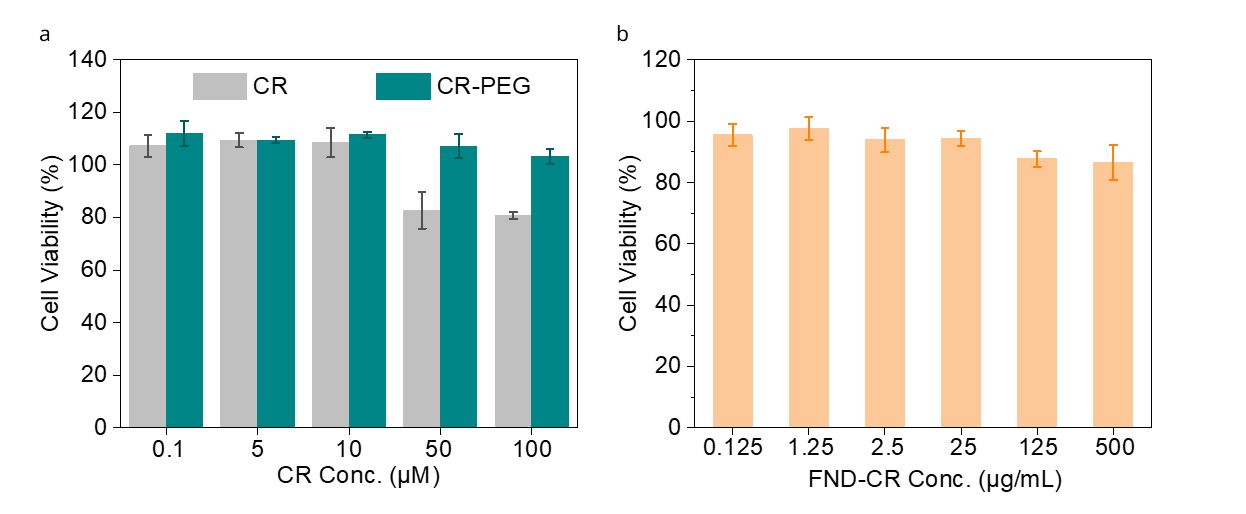


Figure S15. Cytotoxicity of CR, CR-PEG, and FND-CR in J774A.1 macrophages**.** J774A.1 macrophage-like cells were cultured in complete DMEM medium and treated with varying concentrations of CR, CR-PEG (**a**), and FND-CR nanoparticles (**b**) for 24 hours. Cell viability was measured using the CellTiter-Glo® luminescence assay. All three compounds exhibited minimal cytotoxicity across tested concentrations, confirming good cytocompatibility under the applied experimental conditions. Data represent mean ± SD from 3 replicates.


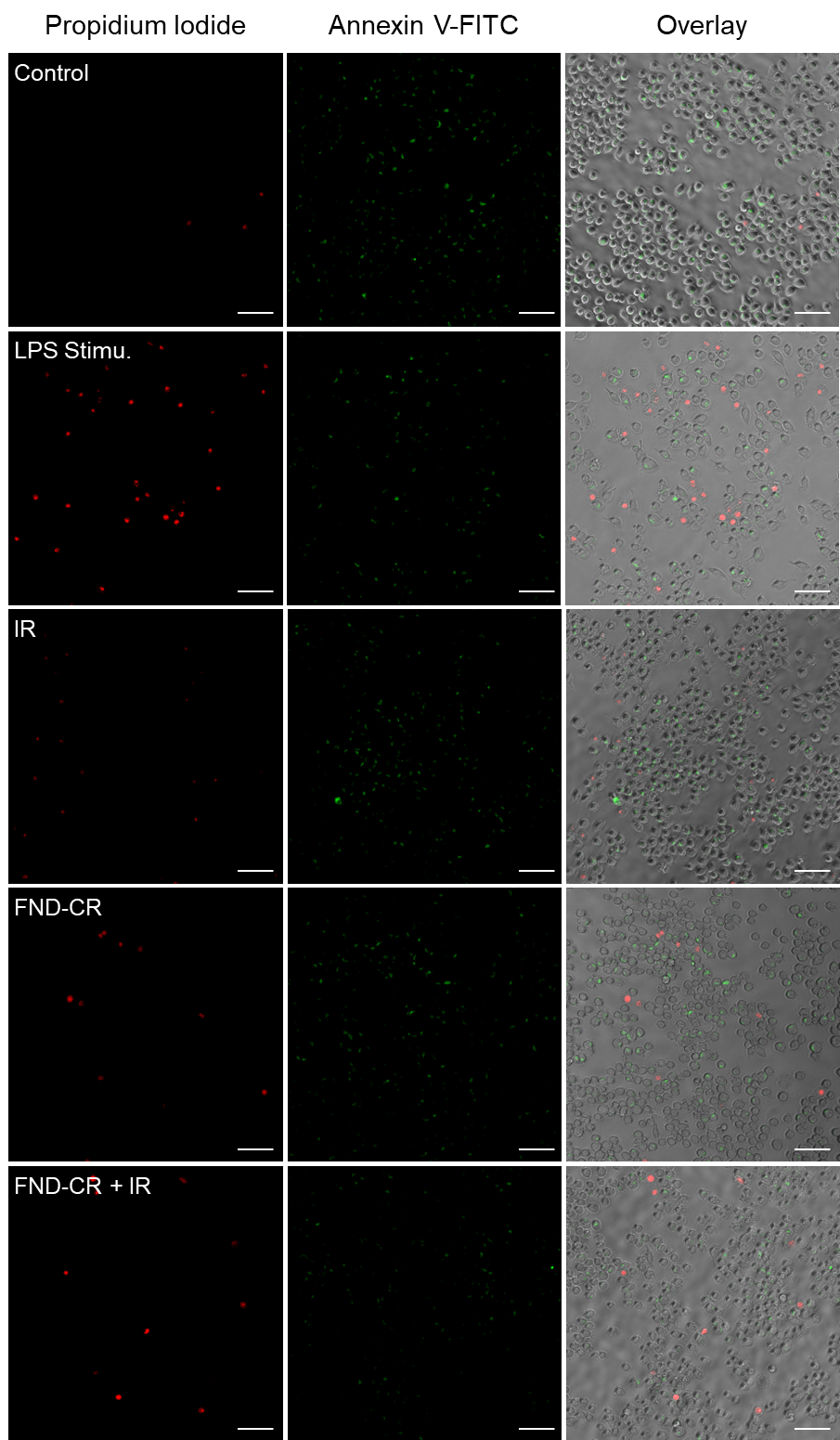


Figure S16. Early apoptosis and cell necrosis assays with Annexin V-FITC and propidium iodide staining, respectively. J774A.1 cells were incubated with FND-CR (30 μg/mL) and irradiated for 15 min using a near-infrared (NIR) LED lamp (810 nm lamp; 0.6 W/cm^2^). Cells treated with LPS, only NIR irradiation, or only FND-CR were also investigated. Cells without any treatment were set as the negative control. Green and red represent apoptotic and dead cells, respectively. Scale bar = 100 µm.


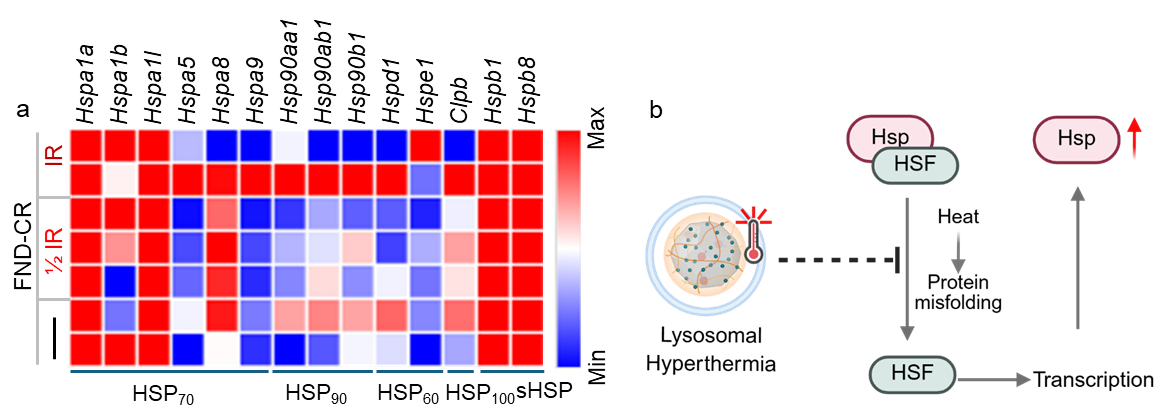


Figure S17.  (a) Transcriptome heatmap showing expression levels of genes from the heat shock protein (HSP) family. Stress-inducible cytosolic HSP70s include *Hspa1a*, *Hspa1b*, and *Hspa1l*; *Hspa8* encodes the constitutively expressed cytosolic HSC70; *Hspa5* and *Hsp90b1* represent ER-resident HSP70 and HSP90, respectively; *Hspa9*, *Hspd1*, *and Hspe1* are mitochondrial HSPs. *Hsp90aa1* and *Hsp90ab1* encode the inducible and constitutive cytosolic HSP90 isoforms. *ClpB*, a member of the HSP100 family, partners with HSP70 to facilitate the disaggregation of proteins. *Hspb1* and *Hspb8* are small heat shock proteins (HSPs) involved in regulating autophagy and stress responses. FND-CR treatment modestly induced expression of certain HSP70 isoforms and small HSPs. However, additional NIR irradiation (FND-CR + IR) did not result in further upregulation of HSP gene expression. (**b**) Mechanism illustration of how heat shock factors (HSF) sense the heat in the cytoplasm and further lead to HSP overexpression. Current Transcriptome analysis suggests that lysosomal heating alone was insufficient or confined to activate a canonical heat shock transcriptional program.

## Table S3. Functions of Genes listed in Figure 4B in the Manuscript.

| Genes | Functions |
| --- | --- |
| *Nadk* | Supports antioxidant defence by generating NADP⁺ for the detoxification of ROS. |
| *Ncoa7* | Contributes to lysosomal redox buffering; stabilizes lysosomal membranes. |
| *Txnip* | A redox sensor that inhibits thioredoxin; its downregulation suggests enhanced antioxidant activity. |
| *Cflar* | Protects against ROS-induced or lysosomal membrane permeabilization (LMP)-induced apoptosis via caspase-8. |
| *Oaz1* | Modulates polyamine metabolism and cellular redox states. |
| *Gja1* | Mediates ROS-linked calcium signalling. |
| *Plk1* | Its downregulation reflects oxidative stress–induced suppression of cell cycle progression. |
| *Rab32* | Regulates lysosome-related organelle biogenesis. |
| *Pxylp1* | Contributes to extracellular matrix remodelling and may influence vesicle dynamics. |
| *Snx24* | A sorting nexin involved in endosomal recycling; its downregulation suggests impaired trafficking under stress. |


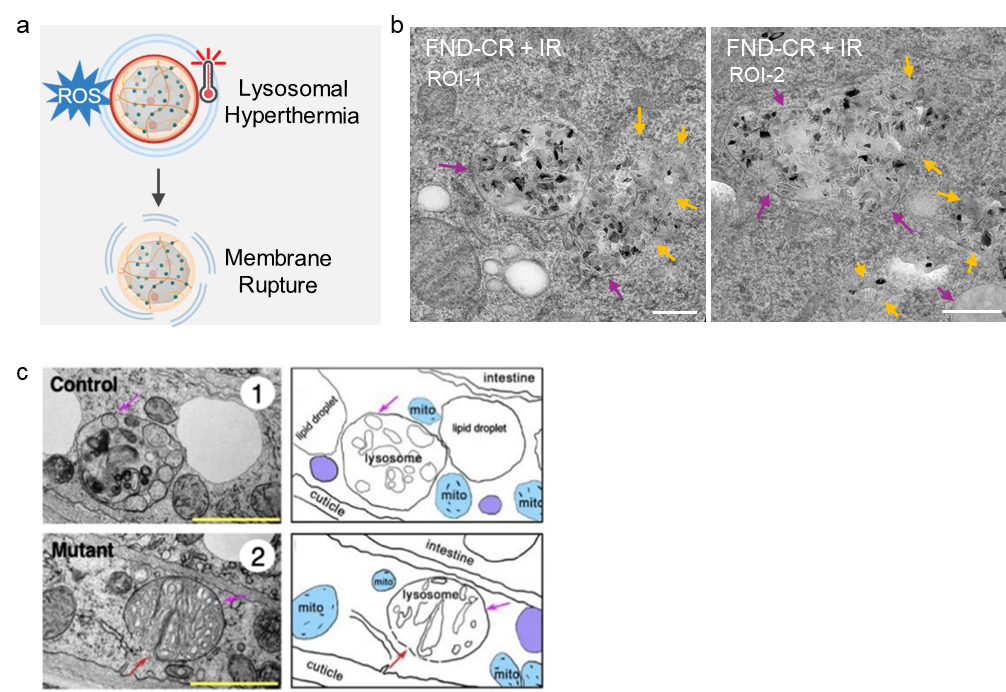


Figure S18. Lysosomal membrane rupture appeared after Irradiation of FND-CR. (**a**) Scheme illustration of lysosomal hyperthermia-induced lysosomal membrane rupture (LMP). (**b**)TEM images of J774A.1 macrophage cell with 15 min NIR irradiation (810 nm, 0.6 W/cm²) following FND-CR uptake. In irradiated cells, the membrane boundaries of FND-CR–containing vesicles appear discontinuous or partially lost, indicating lysosomal membrane rupture (yellow arrows), whereas intact lysosomes exhibit well-defined, continuous membranes (purple arrows). Scale bar: 500 nm. (**c**) Reference TEM image from Yuan Li et al. ^[^*^10^*^]^ showing intact endo-lysosomal membranes as continuous structures (purple arrows) and disrupted lysosomes displaying discontinuous or degraded boundaries (red arrows). Adapted with permission from J. Cell Biol. ^[^*^10^*^]^.

**
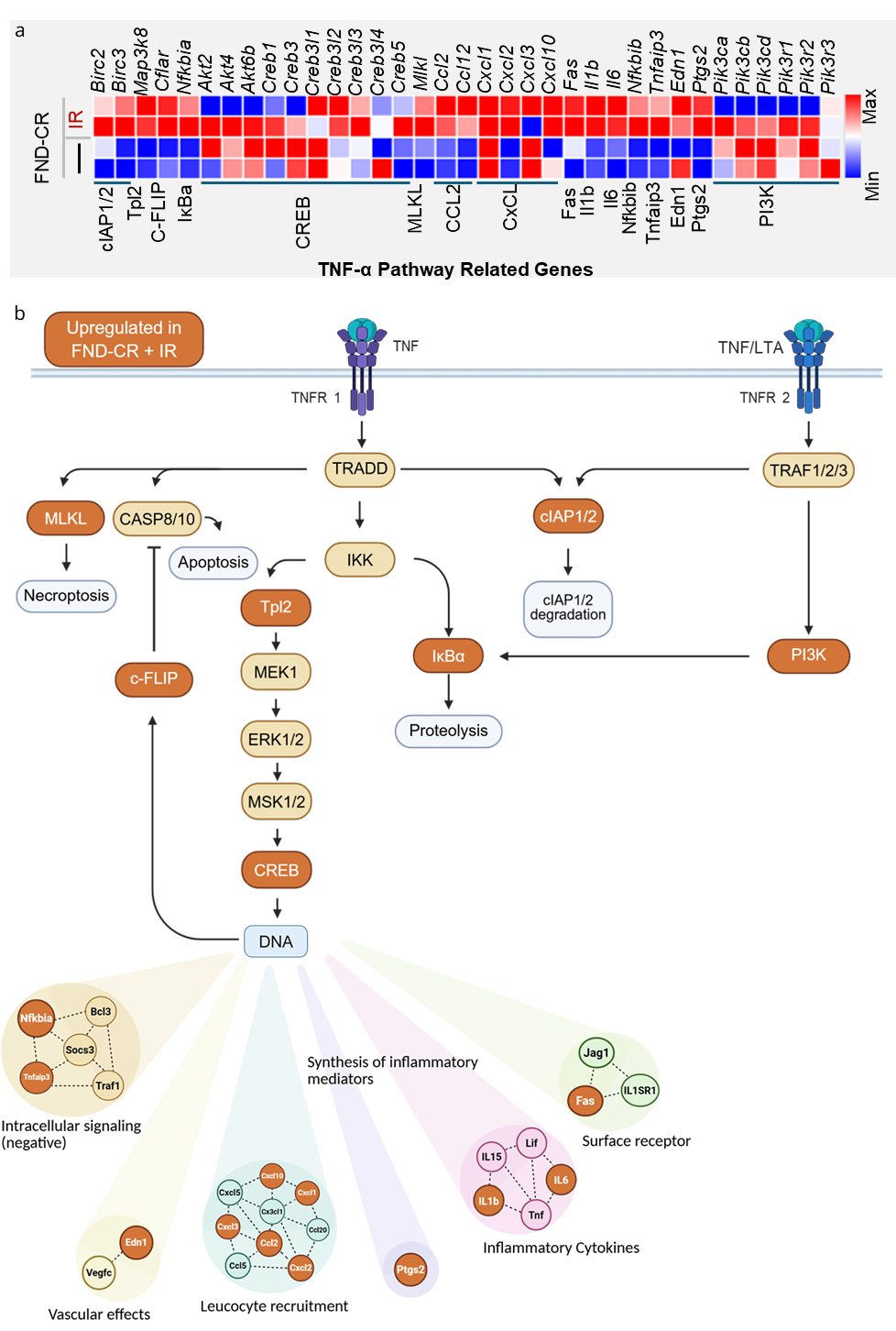
**

Figure S19. TNF pathway enrichment assays. (**a**) Transcriptome heatmap showing expression levels of genes involved in the TNF-α pathway, indicating pathway enrichment following lysosomal hyperthermia. (**b**) Enrichment of TNF-α signalling pathway components following lysosomal heating. Comparative pathway enrichment analysis of FND-CR + NIR irradiation (0.6 W/cm²) versus FND-CR alone. Origin indicates significantly upregulated expression; yellow indicates unchanged expression. Lysosomal heating led to the upregulation of key TNF-associated signaling components, including MLKL (a necroptosis effector), c-FLIP (*Cflar*) (an inhibitor of caspase–8–mediated apoptosis), TPL2 (*Map3k8*) (a kinase linking TNF to ERK activation), and CREB1 (a stress-responsive transcription factor). In addition, IκBα (*Nfkbia*) and cIAP1/2 (*Birc2/3*)—regulators of NF-κB signaling and cell survival—were elevated, alongside PI3K subunits, indicating coordinated activation of pro-survival and inflammatory responses. These findings suggest that lysosome-confined stress engages a noncanonical TNF-like transcriptional program.


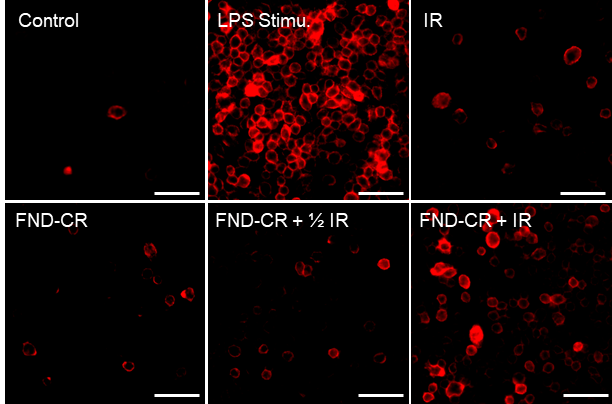


Figure S20. Representative fluorescence images of anti-CD80-PE and antiCD86-PE stained macrophages after the different treatments: no treatment (control), LPS stimulation (positive control), NIR irradiation only (0.6 W/cm², 15 min), FND-CR treatment, half NIR irradiation (0.3 W/cm², 15 min), and NIR irradiation (0.6 W/cm², 15 min) on FND-CR. Scale bar = 50 µm.


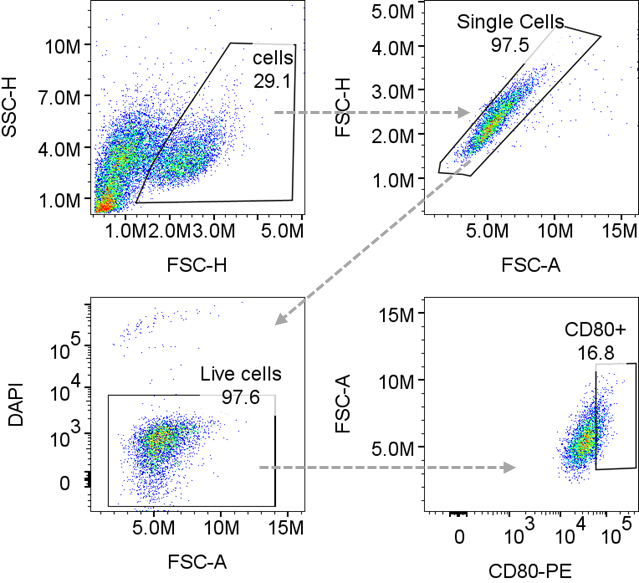


Figure S21. Gating example of flow cytometry analysis. Here, J774A.1 cells were incubated with FND-CR (30 μg/mL) and irradiated for 15 min using a near-infrared (NIR) LED lamp (810 nm lamp; 0.6 W/cm²), and stained with CD80-PE antibody was used as an example. The cells were first distinguished by both forward scatter (FSC) and side scatter (SSC). Then, single cells were selected based on the cellular size according to FSC. Live cells can be selected by the DAPI staining. Then, CD80-positive cells in certain groups can be identified by comparing them with the control group. All the flow cytometry data in the study were processed using the same gating settings. CD80 or CD86-positive cells were determined by their respective control groups, where cells were stained with CD80-PE or CD86-PE antibodies without any treatment.


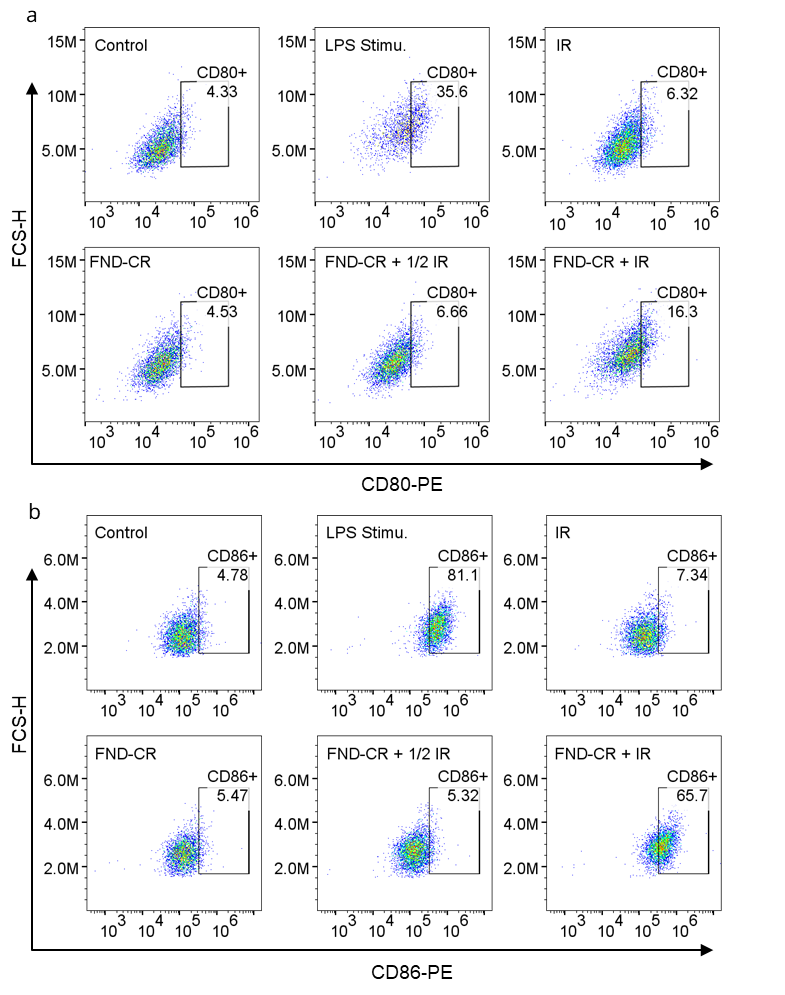


Figure S22. (a, b) Flow cytometry analysis of J774A.1 after various treatments: no treatment (control), LPS stimulation (positive control), NIR irradiation only (0.6 W/cm², 15 min), FND-CR treatment, half NIR irradiation (0.3 W/cm², 15 min), and NIR irradiation (0.6 W/cm², 15 min) on FND-CR, using consistent gating settings. Data are presented as scatter plots of forward scatter height (FSC-H) versus fluorescence intensity of anti-CD80-PE (**a**) or anti-CD86-PE (**b**).


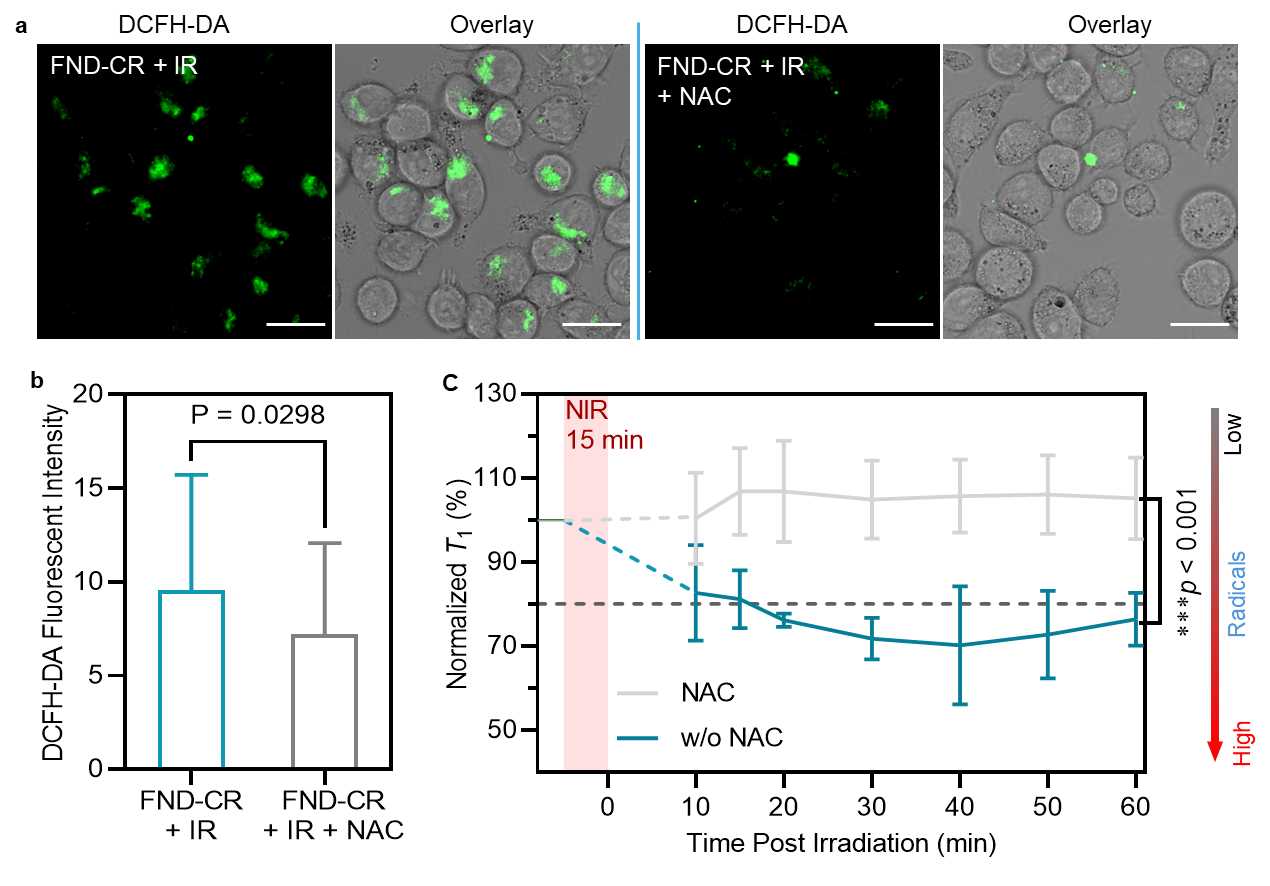


Figure S23. Intracellular oxidative stress assay using NAC as a ROS scavenger. J774A.1 macrophages were incubated with FND-CR (30 μgmL) for 20 h. Before NIR irradiation, cells were pretreated with 10 mM N-acetyl-L-cysteine (NAC) for 30 min and maintained in 5 mM NAC during irradiation (810 nm, 0.6 W/cm², 15 min). (**a**) Fluorescence images of DCFH-DA–stained J774A.1 cells (10 μM, 30 min, serum-free medium). Scale bar = 20 μm. (**b**) Quantification of DCFH-DA fluorescence intensity from single-cell analysis (n = 50). Data are presented as mean ± SD. (**c**) *In-situ* radical sensing via time-resolved *T*₁ relaxometry on individual FND-CR particles. *T*₁ relaxation was recorded continuously for 10 min before irradiation, paused during irradiation (15 min), and resumed afterward to monitor intracellular radical dynamics. A decrease in *T*₁ indicates increased local radical generation. Data are presented as mean ± SD (n = 4). Statistical significance was assessed using one-way ANOVA (*** *p* < 0.001).


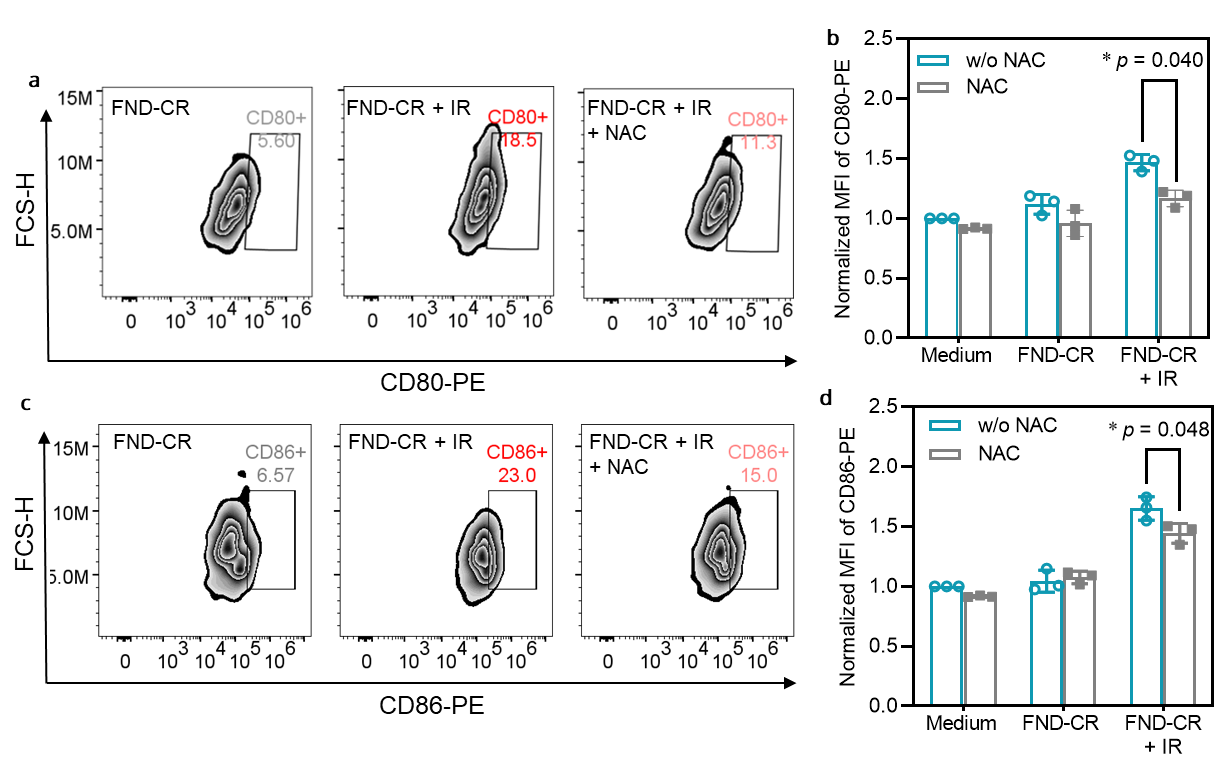
Figure S24. Flow cytometry analysis of NAC-treated J774A.1 cells. J774A.1 cells incubated with FND-CR (30 μg/mL) for 20 h. Prior to NIR irradiation, cells were pretreated with 10 mM NAC for 30 mins and maintained in 5 mM NAC during NIR irradiation (810 nm; 0.6 W/cm^2^, 15 min). After another 20 h incubation, J774A.1 cells were stained with anti-CD80-PE (**a**, **b**) or anti-CD86-PE (**c**, **d**), and analyzed by flow cytometry. (**a**, **c**) Representative zebra plots of forward scatter height (FSC-H) versus fluorescence intensity of anti-CD80-PE or anti-CD86-PE. (**b**, **d**) Quantification of mean fluorescence intensity (MFI) of CD80 and CD86 expression, normalized to the untreated control group (n = 3). Data are presented as mean ± SD. Statistical significance was assessed using an unpaired *t-test*, with *p*-values indicated.


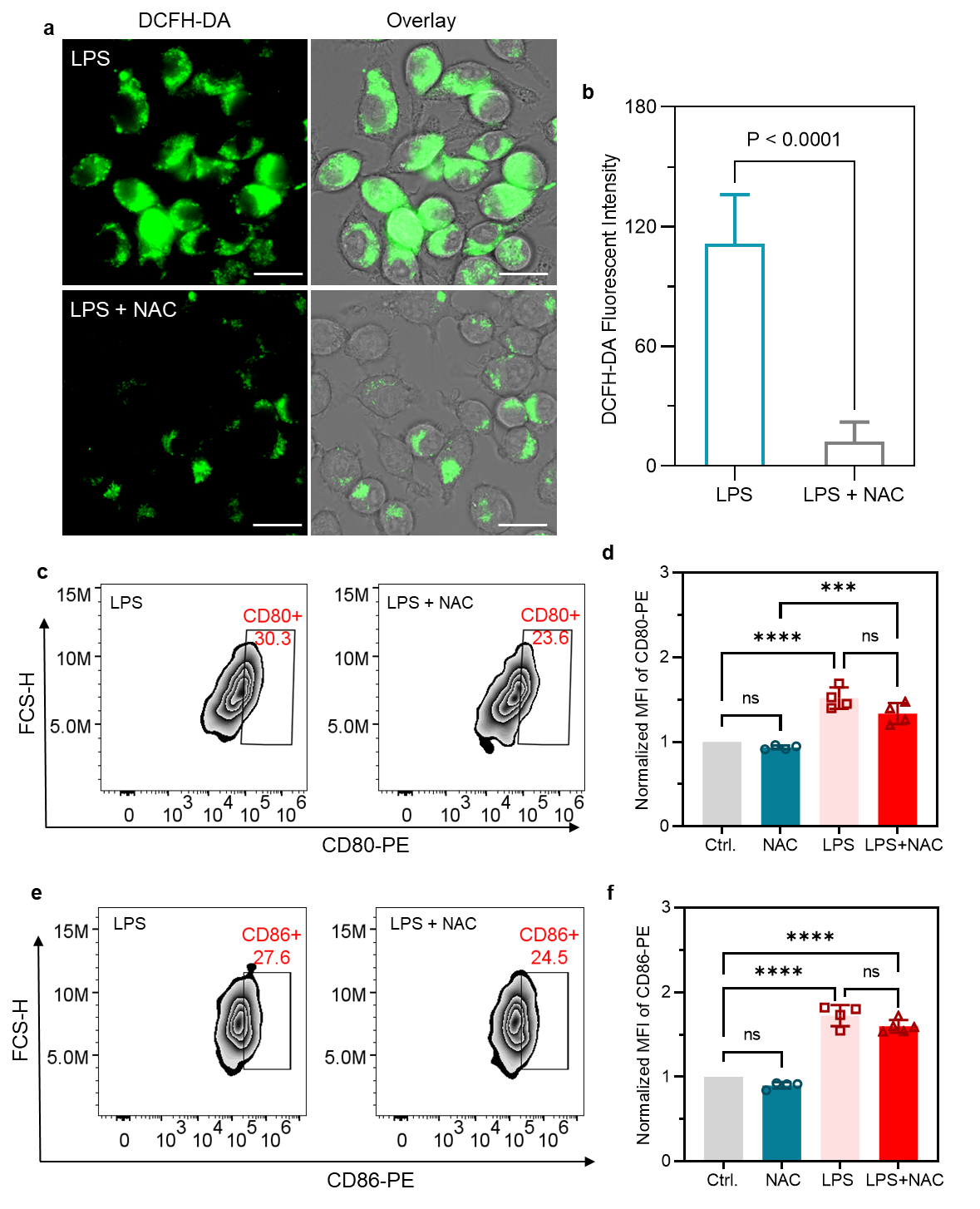


Figure S25. Validation of NAC-mediated ROS scavenging in LPS-stimulated J774A.1 macrophages. (**a**, **b**) Intracellular oxidative stress assay in J774A.1 cells treated with LPS or co-treated with LPS and NAC. Before LPS stimulation, cells were pretreated with 10 mM NAC for 30 min, followed by co-incubation with 1x LPS and 5 mM NAC for 2 h. Cells were then stained with 10 μM DCFH-DA in serum-free medium for 30 min and imaged using a fluorescence microscope. Scale bar = 20 μm (**a**). (**b**) Quantification of DCFH-DA fluorescence intensity from single-cell analysis (n = 50). Data are presented as mean ± SD. Statistical significance was determined using a two-tailed unpaired *t-test* (*p* < 0.0001). (**c**-**f**) Flow cytometry analysis of NACmediated LPS-stimulated J774A.1 macrophages. After another 20 h incubation post the 1x LPS or 1x LPS + NAC (5 mM) treatment, J774A.1 cells were stained with anti-CD80-PE (**c**) or anti-CD86-PE (**f**) and analyzed by flow cytometry. (**d**, **e**) Quantification of mean fluorescence intensity (MFI) for CD80 (**d**) and CD86 (**e**), normalized to untreated controls (n = 4). Data are presented as mean ± SD. Statistical significance was determined using an unpaired *t-test* (**p* < 0.05, ***p* < 0.01, ****p* < 0.001, *****p* < 0.0001).


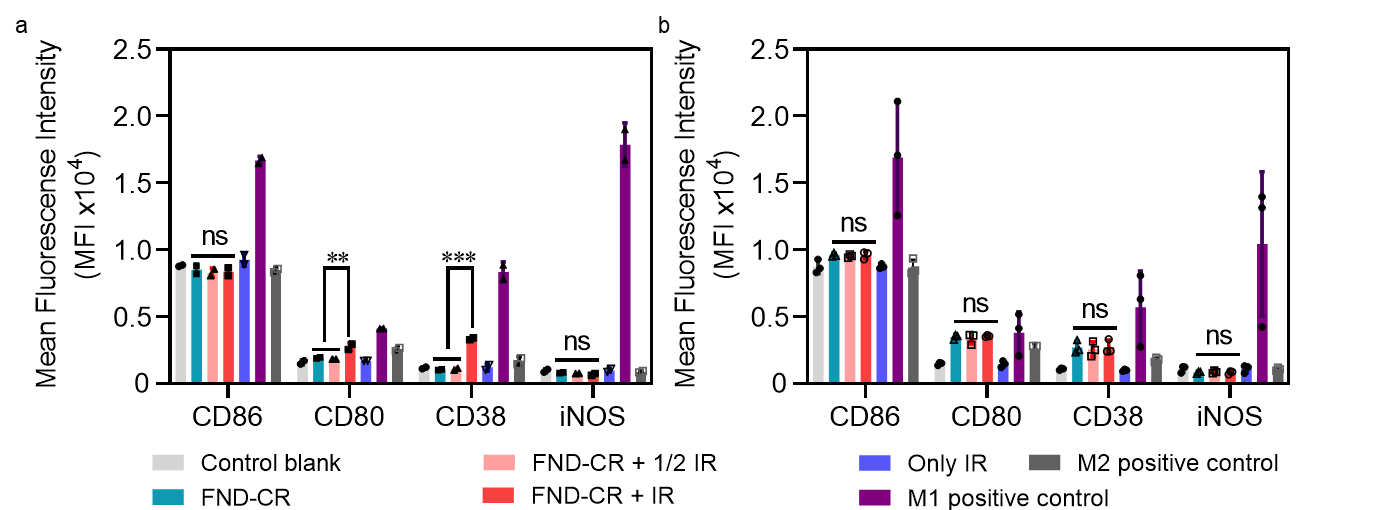


Figure S26. Flow cytometry analysis of primary bone marrow–derived macrophages (BMDMs) treated with FND-CR and subjected to NIR irradiation. BMDMs were incubated with FND-CR (50 μg/mL) for 20 h, followed by irradiation with 810 nm NIR light at either full power (0.6 W/cm², IR) or half power (0.3 W/cm², ½ IR) for 20 min. After an additional 48 h incubation, cells were stained with anti-CD80, anti-CD86, anti-CD38, and anti-iNOS antibodies and analyzed by flow cytometry. The data represent two replicates of two or three independent biological replicates. (**a**) Representative dataset showing clear upregulation of M1-associated surface markers under IR conditions, derived from two mice. (**b**) Representative dataset showing weak or absent polarization response (derived from three mice), illustrating the variability observed among biological replicates. Data are presented as mean ± SD. Statistical significance was determined using two-way ANOVA followed by Tukey’s multiple comparisons test (**p* < 0.05, ***p* < 0.01, ****p* < 0.001, ns, not significant).


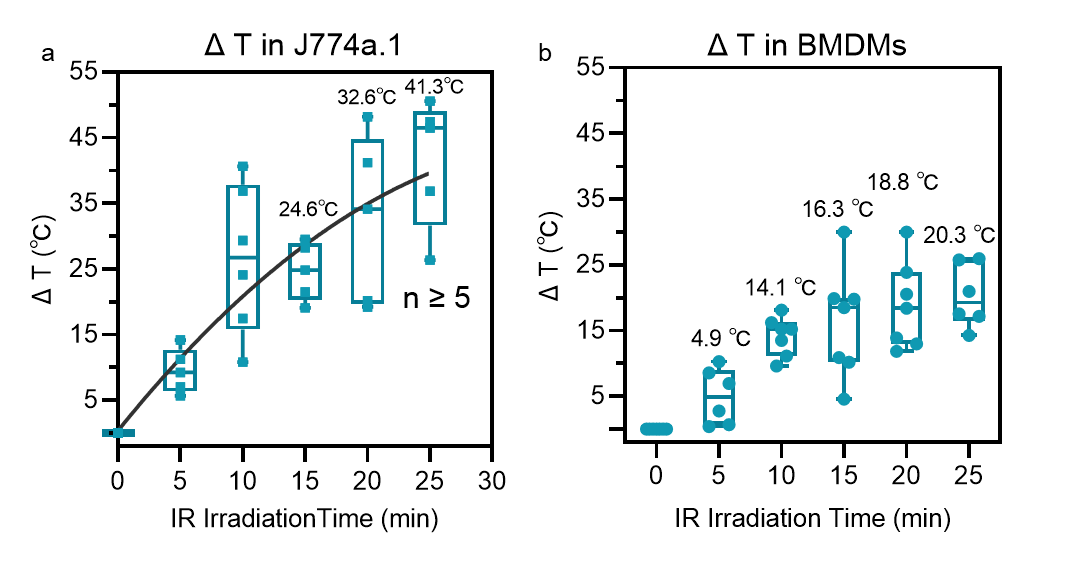


Figure S27. Intracellular temperature change readout in J774A.1 (a) and BMDMs (b) from ODMR measurements on individual FND-CR particles under NIR irradiation (810 nm, 0.6 W/cm²) for 25 minutes (n ≥ 5).

# References

1. R. E. Heap *et al.*, Proteomics Characterisation of the L929 Cell Supernatant and Its Role in Bmdm Differentiation. *Life Sci. Alliance*, **2021**, 4, e202000957.

2. T. T. Bai, N. Gu, Micro/Nanoscale Thermometry for Cellular Thermal Sensing. *Small*, **2016**, 12, 4590-4610.

3. H. S. Jung *et al.*, Organic Molecule-Based Photothermal Agents: An Expanding Photothermal Therapy Universe. *Chem. Soc. Rev.*, **2018**, 47, 2280-2297.

4. H. T. Sun *et al.*, Near-Infrared Photoactivated Nanomedicines for Photothermal Synergistic Cancer Therapy. *Nano Today*, **2021**, 37, 101073.

5. Y. Yue, X. Wang, Nanoscale Thermal Probing. *Nano Rev.*, **2012**, 3, 11586.

6. J. J. Zhou, B. del Rosal, D. Jaque, S. Uchiyama, D. Y. Jin, Advances and Challenges for Fluorescence Nanothermometry. *Nat. Methods*, **2020**, 17, 967-980.

7. K. Okabe, R. Sakaguchi, B. Shi, S. Kiyonaka, Intracellular Thermometry with Fluorescent Sensors for Thermal Biology. *Pflugers Arch.*, **2018**, 470, 717-731.

8. G. Kucsko *et al.*, Nanometre-Scale Thermometry in a Living Cell. *Nature*, **2013**, 500, 54-58.

9. L. D. Far, M. D. Dramicanin, Luminescence Thermometry with Nanoparticles: A Review. *Nanomaterials*, **2023**, 13, 2904.

10. Y. Li *et al.*, The Lysosomal Membrane Protein Scav-3 Maintains Lysosome Integrity and Adult Longevity. *J. Cell Biol.*, **2016**, 215, 167-185.
